# Enhancing Interface Connectivity for Multifunctional Magnetic Carbon Aerogels: An In Situ Growth Strategy of Metal‐Organic Frameworks on Cellulose Nanofibrils

**DOI:** 10.1002/advs.202400403

**Published:** 2024-03-14

**Authors:** Jing Qiao, Qinghua Song, Xue Zhang, Shanyu Zhao, Jiurong Liu, Gustav Nyström, Zhihui Zeng

**Affiliations:** ^1^ Key Laboratory for Liquid‐Solid Structural Evolution and Processing of Materials, School of Materials Science and Engineering Shandong University Jinan 250061 P. R. China; ^2^ School of Mechanical Engineering Shandong University Jinan 250061 P. R. China; ^3^ Laboratory for Building Energy Materials and Components Swiss Federal Laboratories for Materials Science and Technology (Empa) Dübendorf 8600 Switzerland; ^4^ Laboratory for Cellulose and Wood Materials Swiss Federal Laboratories for Materials Science and Technology (Empa) Dübendorf 8600 Switzerland; ^5^ Department of Health Sciences and Technology ETH Zürich Zürich 8092 Switzerland

**Keywords:** aerogel, cellulose nanofibril, in situ growth, metal–organic framework, multifunctional

## Abstract

Improving interface connectivity of magnetic nanoparticles in carbon aerogels is crucial, yet challenging for assembling lightweight, elastic, high‐performance, and multifunctional carbon architectures. Here, an in situ growth strategy to achieve high dispersion of metal–organic frameworks (MOFs)‐anchored cellulose nanofibrils to enhance the interface connection quality is proposed. Followed by a facile freeze‐casting and carbonization treatment, sustainable biomimetic porous carbon aerogels with highly dispersed and closely connected MOF‐derived magnetic nano‐capsules are fabricated. Thanks to the tight interface bonding of nano‐capsule microstructure, these aerogels showcase remarkable mechanical robustness and flexibility, tunable electrical conductivity and magnetization intensity, and excellent electromagnetic wave absorption performance. Achieving a reflection loss of −70.8 dB and a broadened effective absorption bandwidth of 6.0 GHz at a filling fraction of merely 2.2 wt.%, leading to a specific reflection loss of −1450 dB mm^−1^, surpassing all carbon‐based aerogel absorbers so far reported. Meanwhile, the aerogel manifests high magnetic sensing sensibility and excellent thermal insulation. This work provides an extendable in situ growth strategy for synthesizing MOF‐modified cellulose nanofibril structures, thereby promoting the development of high‐value‐added multifunctional magnetic carbon aerogels for applications in electromagnetic compatibility and protection, thermal management, diversified sensing, Internet of Things devices, and aerospace.

## Graphical abstract

A strategy for in situ growing MOFs on cellulose nanofibrils is proposed to promote interface connectivity. Strong anchoring of MOFs produces a unique structure of nano‐capsules tightly embedded in carbon skeletons. The product CoFe/carbon aerogels exhibit exceptional specific reflection loss of −1450 dB mm^−1^, surpassing all reported carbon aerogels, and demonstrate application potential in thermal management and magnetic sensing.

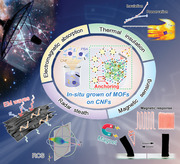

## 1 Introduction

The rapid growth of the Internet of Things (IoT) has created a higher demand for electronic and communication technology. As a result, there is a strong need for high‐performance and multifunctional electronic devices or materials that possess excellent mechanical properties, outstanding electromagnetic compatibility, efficient thermal management, and precise sensing capabilities.^[^ ^1^ ^]^ 3D porous carbons show great promise due to their low density, high stability, adjustable conductivity, and excellent processability.^[^ ^2^ ^]^ Within the realm of 3D porous carbons, polymer‐derived carbons deserve further exploration, as they offer advantages such as adjustable conductivity and a flexible preparation approach that surpass other carbon materials like graphene and carbon nanotubes. Some impressive multifunctional polymer‐derived carbon aerogels have been developed, showcasing significant potential for various applications.^[^ ^3^ ^]^ Cellulose, the most abundant renewable and environmentally friendly polymer on Earth, holds promise as a carbon source to reduce reliance on petrochemical polymers. It is considered a highly attractive component for the future materials industry.^[^ ^4^ ^]^ Moreover, when cellulose is prepared in its nanomaterial form, cellulose nanofibrils (CNFs), the abundant functional groups (such as –OH and –COOH) allow them to exhibit high compatibility and strong interaction with diverse nanoparticles, including metals,^[^ ^5^ ^]^ transition metal carbides/nitrides (MXenes),^[^ ^6^ ^]^ and metal–organic frameworks (MOFs).^[^ ^7^ ^]^ Accordingly, their carbon derivatives are also endowed with a high potential for achieving various functions.

Loading MOFs into cellulose is highly competitive. In addition to the component diversities and tunable topological microstructure of MOFs, one of the main reasons is that their electro‐magnetic coupling carbonized derivative products can be utilized in diverse application scenarios, including electric induction/sensing,^[^ ^8^ ^]^ electromagnetic compatibility,^[^ ^9^ ^]^ and other functionalities.^[^ ^10^ ^]^ Assembling pore‐designed MOF/CNF composite aerogels using bottom‐up approaches for specified functions has become a rational choice for materials synthesis.^[^ ^11^ ^]^ However, the mainstream “ex‐situ” method of mechanically mixing MOF particles with CNFs still prevails in constructing nanocomposites,^[^ ^7^, ^12^ ^]^ leading to the unresolved issue of MOFs agglomerating in high loading conditions and resulting in particle–matrix interface damage. The poor interface quality causes unavoidable degradation in mechanical properties or other functionalities.^[^ ^13^ ^]^ Recent attempts of in situ growing MOFs on preformed cellulose aerogels have been made to address particle dispersion issues,^[^ ^14^ ^]^ they still face challenges in achieving a strong particle–matrix interface connection quality. Because the MOF particles can only be weakly attached onto the surface of CNF walls, preventing their carbonized derivatives becoming magnetic carbon aerogels with good interface connectivity. Therefore, there is a pressing need to develop a facile, sustainable, and scalable manufacturing strategy for constructing MOF/cellulose nanocomposite aerogels that can simultaneously achieve high particle dispersion and desirable interface connectivity, enabling the preparation of high‐performance, multifunctional magnetic carbon aerogels.

Herein, we demonstrate a facile in situ strategy to significantly improve the interface connectivity of Prussian blue analogue (PBA) particles on CNF‐based aerogels. Compared to other MOFs, the PBA derived magnetic particles, taking additional advantages of higher metal content, easily‐regulated metal species, and smaller particle size, are more favorable for promoting magnetization and electromagnetic compatibility.^[^ ^15^ ^]^ Combined with the unidirectional freeze‐casting followed by carbonization treatment, low‐density and flexible biomimetic porous magnetic carbon aerogels with high particle dispersion and strong interface connection were produced. The utilization of a unidirectional ice template imparts the aerogels with biomimetic ordered pores to build an interconnected stratified structure, which can strengthen the intrinsically weak connections between carbon lamellas to improve flexibility, elasticity, and toughness.^[^ ^16^ ^]^ The high dispersion and strong anchoring of the PBA particles create a unique architecture in which the CoFe nano‐capsules are tightly embedded and evenly dispersed in the carbon skeleton. This arrangement enhances the interface connection quality, thereby effectively enhancing the mechanical and electromagnetic properties. By adjusting the growth amount of PBA particles, the conductivity, magnetism, and electromagnetic wave absorption (EWA) properties of the CoFe/carbon aerogels can be easily tailored. The carbon aerogels achieve an excellent reflection loss (RL) of −70.8 dB and a broadened effective absorption bandwidth (EAB) of 6.0 GHz at an ultralow filling ratio of merely 2.2 wt.%. This results in a superior specific RL value of −1450 dB mm^−1^, surpassing all carbon‐based aerogel absorbers so far reported. Meanwhile, the aerogels with high electric/magnetic response capability and low thermal conductivity accomplish functions of magnetic sensing, thermal insulation, and infrared stealth technologies, showcasing large application potential in the fields of electromagnetic protection, thermal management, IoT electronic devices, and aerospace technologies. Moreover, the unique in situ growth strategy of MOFs on CNFs can be easily extend to guide the synthesis of other related materials.

## 2 Result and Discussion

**Figure 1:**
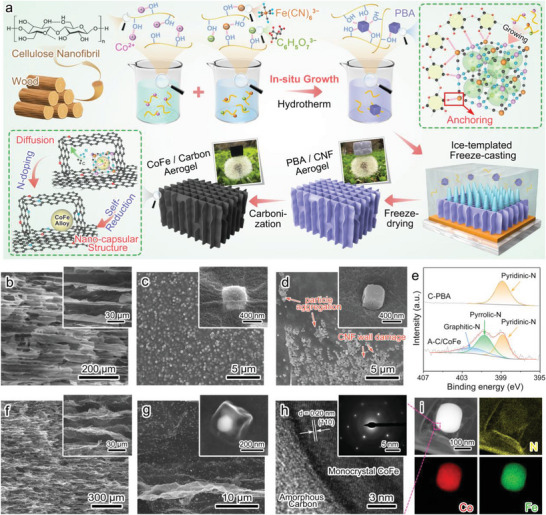
Synthesis illustration and microstructures. a) Schematic showing the process of in situ growth of PBA on CNF for preparing CoFe/carbon biomimetic ordered aerogels. b) SEM images of biomimetic ordered porous PBA/CNF aerogels. c) SEM images of uniform dispersion and high interface connection quality of PBA on CNF‐based cell walls. d) SEM images of agglomerated PBA particles on CNF‐based cell walls and week interface connection for aerogels prepared via a common ex situ mechanical mixing process. e) XPS spectra of C‐PBA and CoFe/carbon aerogels at N 1s region. f) SEM image of biomimetic ordered porous CoFe/carbon aerogels. g) SEM image of CoFe nano‐capsules and their highly uniformed dispersion on carbon walls. h) HR‐TEM image and SAED pattern of CoFe nano‐capsules. i) Element mapping images of CoFe nano‐capsules.

The fabrication process of CoFe nano‐capsule embedded carbon aerogels includes the typical hydrothermal reaction for in situ synthesis of MOF on CNF, followed by ice‐templated freeze‐casting and carbonization process (**Figure** **1**a). Thanks to the numerous hydroxyl/carboxyl groups on the CNFs, the ions used for synthesizing PBA can directly bind to the CNFs by complexation.^[^ ^17^ ^]^ In the hydrothermal treatment, the metal ions on the CNFs coordinate with ferrocyanide ions to form complexes, thus the PBA nuclei are anchored on the CNFs to continuously grow into particles. The combination based on complexation between CNFs and PBA can be confirmed by the peak shift of associated hydroxyl (–OH) in the Fourier transform infrared (FT‐IR) spectra (Figure S1, Supporting Information). Due to the in situ nucleation and growth, the PBA particles can be equably distributed in the CNF dispersion. The introduction of PBA slightly enhances the stability of the dilute CNF dispersion, and the PBA anchored CNF dispersion forms a jelly‐like gel at higher concentration (Figure S2, Supporting Information). Zeta potential measurements indicate that the dispersion stability is maintained with slightly increased zeta potential values for increasing PBA content (Figure S3, Supporting Information), which might be attributed to the negatively charged PBA particles facilitating the dispersion of CNFs. For the maximum PBA proportion of this work (37.3 wt.%), the Zeta potential for composite dispersion is up to −43.4 mV, implying high colloidal stability. Especially, compared with the ex situ method of mixing PBA particles into CNF dispersion, the PBA anchored CNF dispersion exhibits a lower Zeta potential value (Figure S3, Supporting Information), revealing the advantages in improving charge assisted particle dispersion with the in situ method. Besides, the presence of PBA gives the liquid a homogenous bluish‐purple color, further showing the stability of the PBA anchored CNF dispersion.

In the freezing process, unidirectional ice crystals act as templates to repel the PBA/CNF hybrids from the solidification ice front, which makes them accumulate between the ice crystals to form directional pore walls.^[^ ^18^ ^]^ Thus, after freeze‐drying, an anisotropic cellular structure can be achieved in the PBA/CNF aerogels. In the scanning electron microscopy (SEM) image (Figure 1b), oriented channels with an average gap of ≈45 µm can be observed. The PBA nano‐cubes with an edge length of ≈400 nm are found to be covered by overlapping CNFs, which shows that the PBA particles are efficiently embedded inside the CNF walls with strong interactions between the two particles. In other words, the PBA particles are uniformly dispersed in the oriented cell walls with excellent interface connectivity (Figure 1c). In contrast, for the aerogel assembled by an ex situ mechanical mixing (Figure 1d; Figure S4, Supporting Information), the PBA particles obviously agglomerate together and expose on the surface of the CNF walls, resulting in poor interface connection quality between PBA and CNF in the cell walls. This kind of enhancement cannot achieve in the “conventional” in situ aerogels in which the MOFs nucleate and grow only after the preforming of the CNF skeleton, causing the MOF particles to be only attached to the surface of CNF walls,^[^ ^14a–c^ ^]^ the in situ growth strategy in this work demonstrates enormous advantage to improve the dispersion and interface connectivity by forming the MOFs directly onto individual CNFs before constructing the aerogel framework.

In the X‐ray diffraction patterns (Figure S5a, Supporting Information), the characteristic peaks at 17.5°, 24.9°, and 35.5° of PBA can be found for PBA‐anchored CNF aerogels, further confirming the successful synthesis of PBA in the CNF dispersion. The carbonization process converts the organic skeleton of PBA and the CNF walls into carbon to supply the electronic conductivity for functional attributes. Meanwhile, the cobalt and iron ions are reduced into corresponding metal atoms to form CoFe alloys. Carbonization treatment causes noticeable decrease in volume, density increase from 6–18 to 15–30 mg cm^−3^ (Table S1, Supporting Information). However, the loading of nanoparticles can help to prevent the volume shrinkage of CNF aerogels (Table S2, Supporting Information). The carbonization products are confirmed by X‐ray diffraction patterns, and the characteristic peaks substantiate the presence of CoFe alloys (cubic, No. 49–1568) and amorphous carbon (Figure S5b, Supporting Information). The nitrogen‐containing skeleton (−C≡N−) of PBA leads to nitrogen atom‐doping modification of the carbon. In the X‐ray photoelectron spectroscopy (XPS) spectra (Figure 1e; Figures S6,S7, Supporting Information), three deconvolution peaks can be identified as the graphite‐N (402.2 eV), pyrrole‐N (400.7 eV), and pyridine‐N (398.9 eV) for the CoFe/carbon aerogels.^[^ ^18a^ ^]^ However, only one peak from pyridine‐N (398.9 eV) can be observed for the CoFe@carbon particles obtained by directly carbonizing PBA (C‐PBA). This discrepancy is probably because of an increased carbon source provided by the carbonized CNFs allowing the nitrogen atoms to fully diffuse into the graphite lattice. While the insufficient carbon atoms in C‐PBA force the nitrogen atoms to exist only in the form of pyridine‐N. Sufficient nitrogen‐doping in the graphite lattice can efficiently promote the electron transport and electric conductance.^[^ ^19^ ^]^

Regarding morphology, the oriented pore structure is well retained after carbonization, with the average pore gap shrinking to ≈30 µm (Figure 1f). The cell walls become more bent and wrinkled, and the overlapping‐nanofiber structure is replaced by a smoother nanocarbon surface. PBA particle‐derived CoFe nano‐capsules consist of carbon shells with ≈300 nm edge length and monocrystalline CoFe alloy cores with 100–200 nm in diameter. The nano‐capsules strongly adhere to the carbon walls without distinct boundaries (Figure 1g), showcasing excellent particle–matrix interface connection quality. These capsules still maintain high dispersity and good interface connection quality thereby reducing the carbon lamella cleavage caused by agglomeration. The morphology transition from solid nano‐cubes into nano‐capsules may be attributed to the faster decomposition of CNF compared to PBA particles. The carbon and nitrogen atoms from PBA tend to diffuse outside toward the surrounding CNF‐derived carbon to take the shape of a cavity, while the cobalt and iron atoms will gather up and be reduced into CoFe alloy cores. This morphology transformation cannot occur in the directly‐carbonized C‐PBA or CoFe/carbon aerogel derived from PBA/CNF aerogels prepared via mechanical mixing (Figures S9,S10, Supporting Information) since a homogenous coverage of CNF around the PBAs. The carbon shell can separate the metals to restrain excessive eddy effects, and prevent magnetism deterioration from direct metal oxidation. Even at a higher load content, the biomimetic porous carbon structure and nano‐capsule particle morphology can be well maintained (Figure S11, Supporting Information). In the high‐resolution transmission electron microscopy (TEM) image of the nano‐capsules (Figure 1h), the CoFe alloy cores integrates closely with carbon to form electro‐magnetism coupling to promote interfacial polarization. And the selective electron diffraction pattern reveals that the CoFe alloy cores possess a monocrystal nature, which is favorable for charge volume polarization to promote electrical loss capacity.^[^ ^20^ ^]^ According to the IV‐type N_2_ absorption–desorption isotherm, some tiny pores on the carbon supports are found (Figure S12, Supporting Information). This pore structure mainly results from the imperfection of carbon lattices, which is commonly observed in organic‐derived amorphous carbons.^[^ ^21^ ^]^ These pores can endow carbon aerogels which are, e.g., more efficient for gas absorption and as substrates for catalysis. According to Maxwell–Garnett theories (Section S2, Supporting Information), the air introduced by pores can reduce the effective permittivity to improve electromagnetic compatibility performances.^[^ ^22^ ^]^

**Figure 2:**
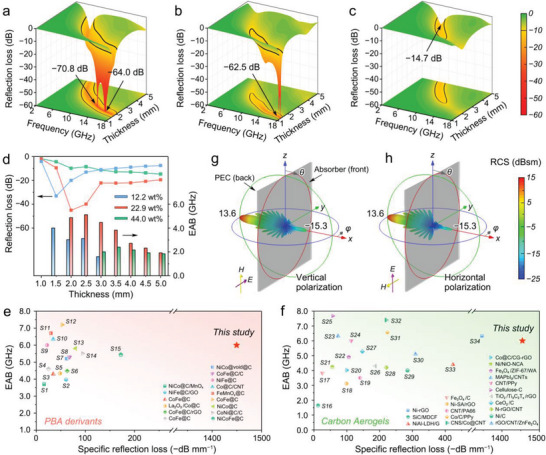
EWA performance and radar stealth simulation. a–c) 3D RL curves of CoFe nano‐capsule embedded carbon aerogel with a) 22.9 wt.%, b) 12.2 wt.%, and c) 44.0 wt.% CoFe alloy contents. d) Statistics of minimum RL values and maximum EAB for CoFe/carbon aerogels. e,f) RCS simulation results of the CoFe/carbon aerogels on a PEC plate under e) vertical polarization and (f) horizontal polarization. g,h) The specific RL value of the PBA‐derived CoFe nano‐capsule embedded carbon aerogels compared with previously reported g) PBA derivatives and h) carbon‐based aerogels (Tables S6 and S7, Supporting Information).

Profiting from the biomimetic ordered porous morphology and excellent interface connectivity, the CoFe/carbon aerogels are featuring excellent EWA performance. Meanwhile, thanks to the low density, the filling rate of the CoFe/carbon aerogels can be as low as ≈2–3 wt.%, superior to that of traditional electromagnetic absorbers. The effective absorption frequency range of the CoFe/carbon aerogel with 22.9 wt.% CoFe content covers 4.3–18 GHz. Two strong absorption peaks of −70.8 dB (2.4 mm, 12.8 GHz) and −64.0 dB (2.0 mm, 15.5 GHz) can be observed, accompanying a maximum EAB of 6.0 GHz (2.3 mm) (**Figure** **2**a). Notably, the EAB of the aerogels at the thickness of 3.0 mm can cover the entire X‐band. This EWA performance outperforms that of pure CNF‐derived carbon aerogel (−11.1 dB of RL and 1.3 GHz of EAB, Figure S13, Supporting Information), and the CoFe/carbon aerogel prepared through “ex‐situ” method (−18.9 dB of RL and 4.1 GHz of EAB, Figure S14, Supporting Information), proving that the magnetic nano‐capsules with high interface connectivity prominently enhances the EWA performance of the carbon. In the top view of the RL representations (Figure S15, Supporting Information), the absorption peak frequency distinctly shifts to a higher range with decreasing matching thickness, and the variation is well in accordance with the quarter‐wavelength matching model (Section S4 and Figure S16, Supporting Information), which implies that the destructive interference plays an essential part in the EWA response.^[^ ^23^ ^]^ Furthermore, the EWA properties of CoFe/carbon aerogels are regulated by changing the loading content of CoFe nano‐capsules. The aerogel with lower CoFe content (12.2 wt.%, Figure 2b) achieves effective absorption in 6.4–18 GHz, with a clearly visible minimum RL value of −62.5 dB (1.4 mm, 17.9 GHz) and a maximum EAB of 4.7 GHz (1.8 mm), while the aerogels with higher CoFe content (44.0 wt.%, Figure 2c) can only get a reduced minimum RL of −14.7 dB (5.0 mm, 6.3 GHz) associated with a decreased EAB of 2.4 GHz (3.3 mm). For a visualized performance comparison, the minimum RL value and corresponding EAB at typical thicknesses are taken out (Figure 2d). Aerogels with 22.9 wt.% CoFe content present superiorities in both absorption intensity and bandwidth within most thicknesses, showcasing that a rational magnetic substance content is of vital importance to gain the best EWA performance.

Achieving strong absorption intensity and broad absorption bandwidth with as low as possible filling rate and minimal thickness is always highly desirable for developing high‐performance EWA materials. Here, the specific RL value (RL value / (thickness × filling rate)) is calculated to evaluate electromagnetic absorption efficiency. Compared with the previously reported PBA derivatives (Figure 2e), the CoFe nano‐capsules embedded carbon aerogels accomplish a remarkable improvement, achieving a specific RL of −1450 dB mm^−1^. Meanwhile, the specific RL value of these aerogels also surpasses all carbon‐based aerogel absorbers so far reported (Figure 2f) due to the strong absorption intensity and low matching thickness. Also, in terms of EAB performance, the CoFe nano‐capsule embedded carbon aerogels demonstrate great performance. These comparisons highlight the major advantages of the CoFe/carbon aerogels as high‐performance EWA materials.

Furthermore, the electromagnetic absorption property of CoFe/carbon aerogels provides them with potential radar stealth capabilities. The radar cross‐section (RCS) is calculated by finite difference time domain method (FDTD) simulation (Tables S4 and S5; Section S5, Supporting Information). In the constructed model, the CoFe/carbon aerogels are attached to a perfect electric conductor (PEC), and far‐field microwaves with horizontal or vertical polarization are radiated toward the aerogels. In the X‐band (10 GHz as an example), the RCS of 22.9 wt.% CoFe/carbon aerogel is below −15 dBsm at a small azimuth angle (|*φ*|≤ 4°), and maintains below −20 dBsm in the azimuth (|*φ*|) of 4–60° (Figure 2g,h). Additionally, the average RCS values are −23.4 dBsm for horizontal polarization and −22.4 dBsm for vertical polarization (Figure S17, Supporting Information). Even for other wavelengths, the RCS of CoFe/carbon aerogel with 22.9 wt.% loading still remains significantly lower than that of the PEC and aerogels with other loading contents (Figures S18–S20, Supporting Information), demonstrating its high radar stealth potential in a wide frequency range.

**Figure 3:**
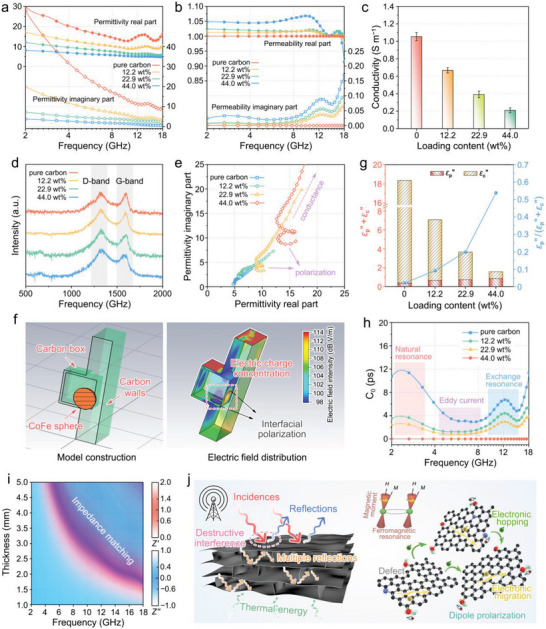
Electromagnetic parameter and EWA mechanism. a–e) The complex permittivity a), complex permeability b), electrical conductivity c), Raman spectra d), Cole–Cole plots e) of pure CNF‐derived carbon and CoFe/carbon aerogels. f) Electric field simulation for a local CoFe@carbon nano‐capsule. g) The contributions of conductivity and polarization, and the proportion of polarization in overall electrical loss. h) *C*_0_ curves of pure CNF‐derived carbon and CoFe/carbon aerogels. i) 2D projection drawings of *Z′* and *Z″* values versus thickness and frequency for 22.9 wt.% CoFe/carbon aerogels. j) Schematic illustrations of EWA mechanisms for the CoFe/carbon aerogels.

Electromagnetic parameters are utilized to understand the performance difference caused by the change in CoFe alloy content loading. Compared with the pure CNF‐derived carbon aerogel, the introduction of CoFe alloy nano‐capsules reduce the complex permittivity (**Figure** **3**a). In contrast, the complex permeability increases with increasing nano‐capsule contents (Figure 3b). Nevertheless, even at a high loading content, the loss tangent of permeability remains much lower than that of the permittivity (Figure S21, Supporting Information), implying that the electrical property plays a leading role in electromagnetic absorption. The ordered interconnected stratified carbon cell walls with a rational graphitization degree induce a non‐negligible conductive loss for electrical consumption. Interestingly, the introduction of high‐conductivity CoFe metals unexpectedly suppress the electrical conductance (Figure 3c). This counter‐intuitive phenomenon can be attributed to the destruction of graphite crystal integrity (irrelevant to the interface connectivity) caused by the carbon consumption during metal reduction. The increasing *I*_D_/*I*_G_ value with nano‐capsule anchoring in Raman spectra (Figure 3d) indicates a higher abundance of carbon crystallite boundaries, suggesting the presence of smaller sp^2^ carbon nanocrystallites and more lattice imperfections. In this case, the fragmented graphite crystals compel the conduction mode of electron migration partially transform into electron hopping, leading to a decline in conductivity. However, the *I*_D_/*I*_G_ values for these aerogels can maintain within the range of ≈1.0–1.2, which means that the graphitization degree of the carbon support is beneficial to balancing conductance attenuation and impedance matching condition to promote electromagnetic absorption.^[^ ^24^ ^]^

Thanks to the good interface connectivity between CoFe nano‐capsules and the carbon skeleton, the interfacial polarization‐relaxation behavior can be greatly promoted, as two conspicuous semicircles in the Cole–Cole plot (Figure 3e and Section S6, Supporting Information) can be observed.^[^ ^25^ ^]^ Here, a simulated electric‐field distribution for a local CoFe@carbon nano‐capsule (Figure S22, Table S8 and Section S7, Supporting Information) reveals an obvious concentration of electric charges at the CoFe/carbon interfaces (Figure 3f), verifying the contribution of interfaces on the electric field attenuation. Here, on‐linear least squares fitting is applied to distinguish the contributions of conductive loss (*ε*_c_ *″*) and polarization loss (*ε*_p_ *″*). As the fitting result (Figure 3g), although the overall dielectric attenuation ability decreases with the loading of CoFe nano‐capsules, the polarization loss continues to rise. Moreover, the proportion of polarization (*ε*_p_ *″*/(*ε*_c_ *″* + *ε*_p_ *″*)) increases from 2.0% (pure carbon) to 53.8% (44.0 wt.% loading). Thus, the introduction of CoFe@carbon nano‐capsules significantly enhances the polarization loss capasity of the CoFe/carbon aerogels. Compared with the aerogels prepared through “ex situ” method (Figure S23, Supporting Information), both the conductive loss and polarization loss of in situ aerogels are much larger, indicating that the improved interface connectivity can effectively improve the electrical attenuation capacity.

The complex permeability increases due to the promoted magnetization saturation from the introduced magnetic CoFe alloys (Figure S24, Supporting Information). The saturation magnetization of the 44 wt.% CoFe/carbon aerogel can be increased to 47.8 emu g^−1^, resulting in considerable magnetic response and attenuation capacity. According to the theories about magnetic loss mechanism (Section S8, Supporting Information), the magnetic losses (Figure 3h) of CoFe/carbon aerogel consist of the natural resonance (2–4 GHz), exchange resonance (8–18 GHz), and eddy current effect (4–8 GHz).^[^ ^18^, ^26^ ^]^ In this study, the superimposed isotherm maps of *Z′* and *Z″* values (*Z′* + *jZ″* = *Z*) were applied to improve accuracy (Figure 3i; Figure S26, and Section S11, Supporting Information), as the common criterion (|*Z*| = |*Z*_in_/*Z*_0_| = 1) would lead to extra meaningless solution. Due to the introduction of highly dispersed CoFe nano‐capsules, a pronounced purple area (where *Z′* ≈ 1 and *Z″* ≈ 0) for the 44.0 wt.% CoFe/carbon aerogel can be observed, indicating excellent impedance matching conditions. Furthermore, the impedance matching area for the 44.0 wt.% CoFe/carbon aerogel is significantly broader than those of other aerogels, demonstrating that the optimized impedance matching conditions contribute to an enhanced EAB performance.^[^ ^27^ ^]^

Based on above discussion, the possible electromagnetic absorption mechanisms are proposed (Figure 3j). The high dispersity and interface connectivity of CoFe nano‐capsules in carbon lamellas, as well as the biomimetic ordered porous morphology optimize the impedance matching conditions, which makes electromagnetic waves more easily enter into the aerogels. Meanwhile, the stratified microstructure induces multiple reflections to extend wave transmission paths promoting its dissipation.^[^ ^28^ ^]^ In addition to the good conductive loss and magnetic loss capability, the improved dispersion and interface connectivity of CoFe nano‐capsules significantly enhances the interfacial polarization‐relaxation, which contributes to the sufficient and diversified electromagnetic attenuation pathways. As a result, the PBA‐derived magnetic CoFe nano‐capsule embedded carbon‐based biomimetic aerogels can achieve excellent electromagnetic absorption performances.

**Figure 4:**
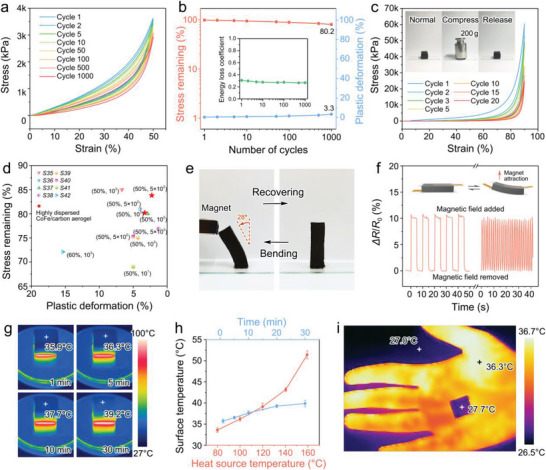
Mechanical property, magnetic sensing, and thermal insulation. a) Compressive stress–strain curves of CoFe/carbon aerogel up to 50% strain for 1000 cycles. b) Residual stress, plastic deformation, and energy loss coefficient of CoFe/carbon aerogel during 1000 cycles at 50% strain. c) Compressive stress–strain curves of CoFe/carbon aerogel up to 90% strain for 20 cycles, and photos of a compression‐release under a 200‐g pressure. d) The stress remaining, and plastic deformation of CoFe/carbon aerogel compared with other polymer‐derived carbon aerogels (Table S9, Supporting Information). e) Photos of reversible bending deformation of CoFe/carbon aerogel induced by a magnet. f) Resistance changes of a typical CoFe/carbon aerogel sensor with a 260 mT magnetic field added and removed. g) Infrared thermal images of CoFe/carbon aerogels heated by a metallic heating platform of 100 °C. h) Surface temperature of 12‐mm thickness CoFe/carbon aerogel after a 10 min heating on platform with various temperatures, and the surface temperature changes on a 100 °C heating platform within 30 min. i) Infrared thermal images of the CoFe/carbon aerogel on hand.

It is typically challenging to achieve satisfying mechanical elasticity for polymer‐derived carbon materials. For the common CoFe/carbon aerogels prepared through “ex situ” method, several sudden changes in the compressive process can be observed in the stress–strain curves (Figure S27, Supporting Information), suggesting the formation of macroscopic cracks, indicating low flexibility and high brittleness. After a 10‐cycle of compression‐release process, the permanent deformation is up to 7.2% for the ex‐situ aerogels, implying inferior mechanical elasticity and recovery. However, due to the interconnected stratified porous microstructure and enhanced interface connection quality between particles and carbon walls, the CoFe/carbon aerogel prepared though in situ method is endowed with a remarkable compressibility, elasticity, and fatigue resistance. After being compressed for 1000 cycles at a strain level of 50% (**Figure** **4**a), the 44.0 wt.% CoFe/carbon aerogel maintained over 80.2% of the maximum stress and suffered only 3.3% permanent deformation (Figure 4b), manifesting that the aerogel can tolerate large elastic deformation without significant structural collapse. Meanwhile, due to the excellent interface connectivity coping with the damage to carbon walls, leading to negligible plastic deformation and reversible friction among lamellas, a small change (from 0.30 to 0.26) in the energy loss coefficient is observed upon cycling. Even under a larger strain of 90% (Figure 4c), the permanent deformation is no more than 3.4% for the first cycle, and still restores 77.9% of its initial shape after 20 compression‐release cycles, as well as a maintained stress of 42.8%. Typically, the CoFe/carbon aerogel can approximately recover its original shape after a compression‐release under a 200‐g pressure (over 600 kPa and 95% deformation). Profiting from the ordered porous structure and improved interface connectivity, the CoFe/carbon aerogel are rendered a superior mechanical fatigue resistance over most polymer‐derived carbon aerogels so far reported (Figure 4d).

The small initial elasticity modulus (2.96 kPa), high elasticity, and good electrical conductivity give the aerogels capabilities for low force sensing, making them suitable for potential applications in precise instruments or wearable devices. Specifically, the introduced magnetic metals provide the carbon aerogel with a unique magnetic sensing capability. Typically, the CoFe/carbon aerogel can produce obvious bending deformation when approached by a magnet. And it rapidly recovers to its initial shape after the magnet is removed (Figure 4e). During the bending process, on one side of the aerogel is compressed to shorten the electron transmission paths, thereby reducing the resistance.^[^ ^29^ ^]^ Here, a typical magnetism‐conductance response is exemplified in the form of a resistivity variation signal under a cyclical addition/removal of a 260 mT magnetic field, and a reproducible resistance change of ≈10% can be observed (Figure 4f), which shows reversible and reliable magnetic sensing performance. These carbon aerogels can be used to manufacture various magnetic sensors, such as geomagnetic sensors, magnetic force sensors, and magnetic angle sensors, and exhibit potential application in navigation systems, aerospace, automotive industry, and smart equipment.

In addition, the biomimetic CoFe/carbon aerogel exhibited reliable thermal insulation properties due to the heat conduction obstruction caused by abundant pores. When placed on a metallic heating platform of ≈100 °C, the upper surface temperatures of a 12 mm‐thick CoFe/carbon aerogel can still maintain 39.2 °C after heating for 30 min (Figure 4g). When the heating temperature is raised to 180 °C, the aerogel can still maintain a surface temperature of merely 52 °C (Figure 4h), demonstrating a large potential for thermal protection or thermal shock resistance. Furthermore, the thermal infrared image (Figure 4i) shows that when a piece of the CoFe/carbon aerogel is placed on hands, it can effectively block the thermal radiation from the body, revealing its high usability for infrared stealth. These explorations further extend the application potential of the CoFe nano‐capsule embedded carbon aerogel as a new type of high‐performance and high‐value‐added multifunctional material.

## 3 Conclusion

Facile in situ growth of MOF on nanocellulose is developed to efficiently promote magnetic nanoparticle dispersion and interface connection quality in novel biohybrid materials. This leads to high interface connectivity of PBA‐derived magnetic CoFe nano‐capsules in biomimetic porous carbon‐based aerogels with excellent functional properties. Rational design of the components and biomimetic ordered pores endow the magnetic nano‐capsule‐embedded carbon aerogels with remarkable mechanical flexibility, elasticity and compressibility, controllable electrical conductivity and magnetization intensity, and regulable EWA performance. The aerogels achieve an excellent RL of −70.8 dB and broadened EAB of 6.0 GHz at an ultralow filling ratio of 2.2 wt.%, leading to a superior specific RL value of −1450 dB mm^−1^, surpassing all carbon‐based aerogel absorbers so far reported. Meanwhile, the low‐density aerogels also integrate high electric/magnetic response abilities with low thermal conductivity, accomplishing potential applications in magnetic sensing, thermal insulation, and infrared stealth functionalities. Combined with the facile and extensible fabrication in situ growth methods, the magnetic nano‐capsule/carbon aerogels with high interface connectivity exhibit great and broad application prospects in the field of electromagnetic compatibility and protection, thermal management, diversified sensing, IoT electronic devices and aerospace.

## 4 Experimental Section

### Preparation of CNF Dispersion with PBA In Situ Growth

In a typical process, 0.15 g of cobalt (II) acetate and 0.25 g of trisodium citrate dihydrate were added into 20 mL of deionized water with stirring until the salts were dissolved completely, followed by 20 g of 2,2,6,6‐tetramethyl‐1‐piperidinyloxy (TEMPO) oxidized CNF aqueous dispersion (supplied by Mujingling Biotechnology Ltd., China) added into the solution with violent vibration for 20 min. Similarly, 0.132 g potassium ferricyanide was added into 30 mL of deionized water with stirring to get a homogeneous solution, followed by 30 g of CNF aqueous dispersion added with violent vibration treatment. Whereafter, the two dispersions were quickly mixed with mild stirring for several minutes, and the mixture was rapidly transferred to a glass bottle with a lid, and placed on an 80 °C of water bath for 12 h. The obtained bluish‐purple dispersion was washed and maintained the original dispersion volume. To change the PBA content, multiplicative mass of salts was used in the solution preparation process.

### Fabrication PBA/CNF and CoFe/Carbon Aerogel

The CNF dispersion after PBA in situ growth was poured into a custom‐made Teflon mold with a copper column bottom plate for an unidirectionally freezing under the action of liquid nitrogen. Subsequently, the PBA/CNF aerogel was obtained by freeze‐drying (−60 °C, 3 Pa) treatment for 48 h. Finally, the PBA/CNF aerogel was put in a custom‐made pressure‐tight steel reaction and heated at 650 °C for 2 h to product CoFe/carbon aerogels. The pure CNF‐derived carbon aerogel was prepared without in situ growth of PBA, followed by the same remaining steps. The carbonized PBA (CoFe@carbon particles) was synthesized without CNF by a direct carbonization treatment of PBA nanoparticles.

## Conflict of Interest

The authors declare no conflict of interest.

## Supporting information

Supporting Information

## Data Availability

The data that support the findings of this study are available from the corresponding author upon reasonable request.
