# Supplementary material for: Enhancing Interface Connectivity for Multifunctional Magnetic Carbon Aerogels: An In Situ Growth Strategy of Metal‐Organic Frameworks on Cellulose Nanofibrils
Source: Adv Sci (Weinh). 2024 Mar 14;11(19):2400403. doi: 10.1002/advs.202400403 (PMC11109645; doi:10.1002/advs.202400403)
Supplement: Supplementary file 1 — Supporting Information [file ADVS-11-2400403-s001.pdf]

ADVANCED SCIENCE
Open Access

Supporting Information

for *Adv. Sci.*, DOI 10.1002/advs.202400403

Enhancing Interface Connectivity for Multifunctional Magnetic Carbon Aerogels: An In Situ Growth Strategy of Metal-Organic Frameworks on Cellulose Nanofibrils

Jing Qiao, Qinghua Song, Xue Zhang, Shanyu Zhao, Jiurong Liu*, Gustav Nyström* and Zhihui Zeng**

Supporting Information

Enhancing Interface Connectivity for Multifunctional Magnetic Carbon Aerogels: An In Situ Growth Strategy of Metal-Organic Frameworks on Cellulose Nanofibrils

Jing Qiao, Qinghua Song, Xue Zhang, Shanyu Zhao, Jiurong Liu*, Gustav Nyström*, Zhihui Zeng**

Dr. J. Qiao, X. Zhang, Prof. J. Liu, Prof. Z. Zeng
Key Laboratory for Liquid-Solid Structural Evolution and Processing of Materials, School of Materials Science and Engineering, Shandong University, Jinan 250061, P. R. China
E-mail: jrliu@sdu.edu.cn (Prof. J. Liu), zhihui.zeng@sdu.edu.cn (Prof. Z. Zeng)

Dr. J. Qiao, Prof. Q. Song
School of Mechanical Engineering, Shandong University, Jinan 250061, P. R. China

Prof. Shanyu Zhao
Laboratory for Building Energy Materials and Components, Swiss Federal Laboratories for Materials Science and Technology (Empa), Dübendorf 8600, Switzerland
E-mail: shanyu.zhao@empa.ch (Prof. S. Zhao)

Prof. Gustav Nyström
Laboratory for Cellulose and Wood Materials, Swiss Federal Laboratories for Materials Science and Technology (Empa), Dübendorf 8600, Switzerland
Department of Health Sciences and Technology, ETH Zürich, Zürich 8092, Switzerland
E-mail: gustav.nystroem@empa.ch (Prof. G. Nyström)

This Supporting Information contains:

Section S1: Experimental Section **Page S-3**

Section S2: Maxwell–Garnett theories **Page S-4**

Section S3: Electromagnetic wave absorption performance calculation **Page S-4**

Section S4: Quarter-wavelength matching model **Page S-5**

Section S5: RCS simulation by *Altair FEKO* and *CST Microwave studio* **Page S-5**

Section S6: Debye relaxation theory **Page S-6**

Section S7: Electric-field distribution simulation by *CST Microwave Studio* **Page S-6**

Section S8: Magnetic loss mechanism **Page S-7**

Section S9: Attenuation coefficient **Page S-8**

Section S10: Intrinsic impedance coefficient **Page S-8**

Section S11: Characteristic impedance coefficient **Page S-9**

Section S12: Supplementary Figures (S1-S28) **Page S-9**

Section S13: Supplementary Tables (S1-S9) **Page S-22**

Section S14: Supplementary References **Page S-27**

Section S1: Experimental Section

Materials: 2,2,6,6-tetramethyl-1-piperidinyloxy (TEMPO) oxidized CNF aqueous dispersion (1.0 wt%) was made from coniferous wood bleached sulfate pulp, and purchased from Mujingling Biotechnology Ltd. (China). Cobalt(II) acetate (AR), trisodium citrate dihydrate (AR), and potassium ferricyanide (AR) were purchased from Sinopharm Chemical Reagent Ltd. (China).

Characterization: The stability of different dispersions were measured by a Zeta potential analyzer (Malvern Zetasizer Nano ZS90). The micro-morphology characterization was carried by the field-emission scanning electron microscopy (Hitachi Model SU-70 and JSM-7610F). The micro-structures and element distributions were characterized by a high-resolution transmission electron microscopy (FEI Talos F200x) equipped with an energy disperse spectroscopy. The crystalline structure was characterized by powder X-ray diffraction (DMAX-2500PC). The surface electronic properties were obtained through X-ray photoelectron spectroscopy (Thermo ESCALAB 250XI). The Fourier transform infrared spectra were recorded by an infrared spectrometer (Thermo Scientific Nicolet iS20). The N_2 absorption–desorption isotherm was obtained by a chemisorption analyzer (Micromeritics ASAP 2460). The Raman spectra were recorded by a Raman spectrometer (Horiba LabRAM HR). The hysteresis loops were recorded by a vibrating sample magnetometer (LakeShore7404). The conductivity/resistivity was measured in a four-probe method by the Tonghui test system (TH26011CS). The stress–strain curves were obtained through a universal testing machine (IS-200N). The infrared thermal images were shot by an infrared induction camera (Fotric223s). The electromagnetic parameters in the frequency range of 2–18 GHz were measured by a vector network analyzer (VNA, Agilent PNA N5244A) in the coaxial method.

To measure the electromagnetic parameters, the aerogels were integrally immersed in liquid paraffin by vacuum, subsequently solidified by cooling and cut into annulus (Φ_{in}, 3.04 mm; Φ_{out}, 7.00 mm). Considering the common filler is paraffin, resins, or rubbers with an average density of approximately 1 g/cm^3, the filling rate in this study is calculated by "ρ (aerogel) / (ρ (aerogel) + 1 g/cm^3)". Thus, the filling rate of the CoFe/carbon aerogels in this study is as low as ~2–3 wt%.

Section S2: Maxwell–Garnett theories

According to the Maxwell–Garnett theory, the effective permittivity ($\varepsilon_{\mathrm{eff}}$) of a material consisting of two different components can be expressed by the followed equations:

$$\varepsilon_{\mathrm{eff}}^{\mathrm{MG}} = \varepsilon_1 \frac{(\varepsilon_2 + 2\varepsilon_1) + 2\rho(\varepsilon_2 - \varepsilon_1)}{(\varepsilon_2 + 2\varepsilon_1) - \rho(\varepsilon_2 - \varepsilon_1)} \quad \textbf{Eq. S1}$$

where ε_1 and ε_2 are the permittivity of host and guest components, respectively, ρ is the volume fraction of the guest. In this work, the host and guest components represent the tight CoFe/carbon and the air, permittivity. Thus, the ε_2 is equal to 1.

$$\varepsilon_{\mathrm{eff}}^{\mathrm{MG}} = \varepsilon_1 \frac{(1 + 2\varepsilon_1) + 2\rho(1 - \varepsilon_1)}{(1 + 2\varepsilon_1) - \rho(1 - \varepsilon_1)} < \varepsilon_1 \quad \textbf{Eq. S2}$$

Therefore, the porous structures can reduce the effective permittivity.

Section S3: Electromagnetic wave absorption performance calculation

The reflection loss (RL) in this study is calculated by electromagnetic parameters, and based on the metallic backing model and transmission line theories. The calculation equations are shown as follows:

$$Z_{\mathrm{in}} = Z_0 \sqrt{\frac{\mu_{\mathrm{r}}}{\varepsilon_{\mathrm{r}}}} \tanh\left(\frac{2\pi jfd}{c}\sqrt{\mu_{\mathrm{r}}\varepsilon_{\mathrm{r}}}\right) \quad \textbf{Eq. S3}$$

$$RL = 20\log\left|\frac{Z_{\mathrm{in}} - Z_0}{Z_{\mathrm{in}} + Z_0}\right| \quad \textbf{Eq. S4}$$

where Z_{in} and Z_0 are on behalf of the input impedance and the free space impedance, respectively; ε_{r} and μ_{r} refer to the complex permittivity and permeability; c is the light speed in vacuum; j is the imaginary unit; and d is the absorber matching thickness.

Section S4: Quarter-wavelength matching model

In the quarter-wavelength matching model, the theoretic matching thickness (t_T) can be calculated by the followed equation:

$$t_T = \frac{n\lambda}{4} = \frac{nc}{4f_M\sqrt{|\mu_r||\varepsilon_r|}} \qquad n = 1,3,5... \qquad \textbf{Eq. S5}$$

where λ is the wavelength of electromagnetic waves; c is the velocity of light in vacuum; f_M is the matching frequency; ε_r and μ_r refer to the complex permittivity and permeability, respectively.

In this work, it can be observed (**Figure S16**) that the t_M points were highly in tune with the t_T curves, indicating that the electromagnetic absorption performances obeyed the quarter-wavelength matching model.

Section S5: RCS simulation by Altair FEKO and CST Microwave studio

Model construction and excitation configuration: The width of the perfect electric conductor (PEC) plate was 150.0 × 150.0 mm, and the thickness was 5.0 mm. The thickness of covered carbon aerogel layer or CoFe/carbon aerogel layer is set as the **Table S4**. The position configuration of far fields was "calculate fields in plane wave incident direction". The plane waves of 6 GHz, 10 GHz, and 15 GHz (midpoint of C, X, and Ku band) single frequency were chosen as the excitation source, and the detail electromagnetic parameters are provided in **Table S5**. For the setting of vertical polarization waves, the incident azimuth angles were restricted within the condition of "$-60° \leq \varphi \leq 60°$; $\theta = 90°$". The polarization angles were 0° for the vertical polarization, and 90° for the horizontal polarization. And the polarization mode was linear. The 3D presentation of RCS values for 22.9 wt% CoFe/carbon aerogel was obtained by *CST Microwave studio*, in which the parameter settings are similar to that of *Altair FEKO*.

Section S6: Debye relaxation theory

The polarization for a dielectric material usually refers to the charge migration or dipole orientation along the electric field. In an alternating electromagnetic field, if the charge migration or dipole orientation cannot keep up with the changing frequency of electromagnetic field, a hysteresis effect will occur, which is called electron or dipole polarization–relaxation. With polarization and conductivity both being considered, the permittivity should be expressed as followed equations.

$$\varepsilon' = \frac{\varepsilon_s - \varepsilon_\infty}{1+\omega^2\tau^2} + \varepsilon_\infty \qquad \textbf{Eq. S6}$$

$$\varepsilon'' = \varepsilon_p'' + \varepsilon_c'' = \omega\tau \frac{\varepsilon_s - \varepsilon_\infty}{1+\omega^2\tau^2} + \frac{\sigma}{\omega\varepsilon_0} \qquad \textbf{Eq. S7}$$

where ε_s and ε_∞ refer to the permittivity at electrostatic field and high-frequency limit, ε_0 means the permittivity of free space; ω, τ and σ are the angular frequency, polarization–relaxation time and conductivity, respectively; ε_p'' and ε_c'' represent the polarization loss and conductive loss, respectively.

Due to the nonnegligible conductivity, the original Debye formula should be modified as the followed equation.

$$\left(\varepsilon' - \frac{\varepsilon_s - \varepsilon_\infty}{2}\right)^2 + \left(\varepsilon'' - \frac{\sigma}{\omega\varepsilon_0}\right)^2 = \left(\frac{\varepsilon_s - \varepsilon_\infty}{2}\right)^2 \qquad \textbf{Eq. S8}$$

Thus, in the ε'' *vs.* ε' curves (Cole–Cole plot), the curves would transform into several distorted semicircles with a "tail" extending to the upper right. The semicircles represent polarization–relaxation behaviors, while the "tail" is on behalf of electrical conductance.

Section S7: Electric-field distribution simulation by CST Microwave Studio

The constructed model of a local CoFe@C nano-capsule is shown as **Figure S22**, and the detail simulation parameters are provided in **Table S8**. The excitation is orthogonal alternating electric field and magnetic field to simulate the electric field distribution of an object in a rectangular waveguide cavity.

Section S8: Magnetic loss mechanism

The magnetic loss originates from the interactions between magnetic domains or dipoles with alternating magnetic field. In the high frequency region, the magnetic loss mainly consists of the domain wall rotation, eddy current loss, ferromagnetic resonance (including natural resonance and exchange resonance), *etc*.

The natural resonance frequency can be calculated by the followed equations.

$$2\pi f_r = \gamma H_a \qquad \textbf{Eq. S9}$$

$$H_a = \frac{4|K_1|}{3\mu_0 M_s} \qquad \textbf{Eq. S10}$$

$$K_1 = \frac{\mu_0 M_s H_c}{2} \qquad \textbf{Eq. S11}$$

where f_r is the natural resonance frequency, γ is the gyromagnetic ratio, H_a is the anisotropy energy, K_1 is the anisotropy coefficient, μ_0 is the initial permeability, M_s is the saturation magnetization, and H_c is the coercivity. According to the equations, the natural resonance frequency will shift to higher frequency with the coercivity increasing.

According to Aharoni's theory, the exchange resonance frequency ω_{kn} can be given by the followed equation.

$$\frac{\omega_{kn}}{\gamma} = \frac{C\mu_{kn}^2}{R^2 M_s} + H_0 - \frac{4\pi}{3}M_s + \frac{2K_1}{M_s} \qquad \textbf{Eq. S12}$$

where γ is the gyromagnetic ratio, C is the exchange constant, μ_{kn} is the eigenvalues of the equation $[J'_n(r)]_{r=R} = 0$ with J_n being Bessel's spherical functions, R is the magnetic particle radius, K_1 is the anisotropy coefficient, M_s is the saturation magnetization, H_0 is the external magnetic field. According to the equation, the exchange resonance frequency will shift to lower frequency range with saturation magnetization increasing.

Generally, for soft magnetic metallic materials, the natural resonance usually occurs at 2–10 GHz, and the exchange resonance locates on higher frequency range.

Under the motivation of alternating magnetic field, the induced current turning around the conductor is called eddy current, which transforms magnetic field energy into heat. The presence of eddy current can be judged by C_0 value, which is calculated by the followed equation.

$$C_0 = \mu''(\mu')^{-2} f^{-1} = \frac{2}{3}\pi\mu_0\sigma d^2 \qquad \textbf{Eq. S13}$$

where μ' and μ'' are the real part and imaginary part of permeability, μ_0 is the initial permeability,

f is the frequency, σ is the electrical conductivity, d is the thickness. According to the equation, if the magnetic loss only caused by only eddy current loss, the C_0 values should be constant with the changing frequency.

Section S9: Attenuation coefficient

The attenuation coefficient (α) is calculated by the followed equation.

$$\alpha = \frac{\sqrt{2}\pi f}{c} \times \sqrt{(\mu''\varepsilon'' - \mu'\varepsilon') + \sqrt{(\mu''\varepsilon'' - \mu'\varepsilon')^2 + (\mu''\varepsilon' - \mu'\varepsilon'')^2}} \quad \textbf{Eq. S14}$$

where f is the frequency; c is the light speed in vacuum; ε' and ε'' refer to the real part and imaginary part of permittivity; ε' and ε'' represent the real part and imaginary part of permeability, respectively. The attenuation coefficient of the aerogels in this work is shown in **Figure S25a**.

Section S10: Intrinsic impedance coefficient

The intrinsic impedance coefficient (M_η) is calculated by the followed equation.

$$M_\eta = \frac{2 \times \mathrm{Re}\left[\sqrt{\mu_\mathrm{r}/\varepsilon_\mathrm{r}}\right]}{\left|\sqrt{\mu_\mathrm{r}/\varepsilon_\mathrm{r}}\right|^2 + 1} \quad \textbf{Eq. S15}$$

where Re[x] refers to the real part of x, ε_r and μ_r represent the complex permittivity and permeability, respectively. The attenuation coefficient of the aerogels in this work is shown in **Figure S25b**.

Section S11: Characteristic impedance coefficient

The characteristic impedance coefficient (Z = Z' + jZ") is calculated from the electromagnetic parameters, and calculated by the followed equations.

$$Z = \frac{Z_{\mathrm{in}}}{Z_0} = \sqrt{\frac{\mu_{\mathrm{r}}}{\varepsilon_{\mathrm{r}}}} \tanh\left(\frac{2\pi jfd}{c}\sqrt{\mu_{\mathrm{r}}\varepsilon_{\mathrm{r}}}\right) \quad \textbf{Eq. S16}$$

$$Z' = \mathrm{Re}\left[\frac{Z_{\mathrm{in}}}{Z_0}\right] \quad \textbf{Eq. S17}$$

$$Z'' = \mathrm{Im}\left[\frac{Z_{\mathrm{in}}}{Z_0}\right] \quad \textbf{Eq. S18}$$

where Z_{in} and Z_0 are on behalf of the input impedance and the free space impedance, respectively, ε_{r} and μ_{r} are the complex permittivity and permeability, c is the light speed in vacuum, j is the imaginary unit, d is the absorber matching thickness, Re[x] and Im[x] represent the real part and imaginary part of x, respectively. In the impedance matching isotherm maps, the overlapping region (purple area in this work) can simultaneously satisfy the requirement of Z' close to 1 and Z" close to 0, which means that the impedance of absorbers is extremely close to that of air, thus the electromagnetic waves can totally enter into the absorber to be consumed.

Section S12: Supplementary Figures (S1-S28)

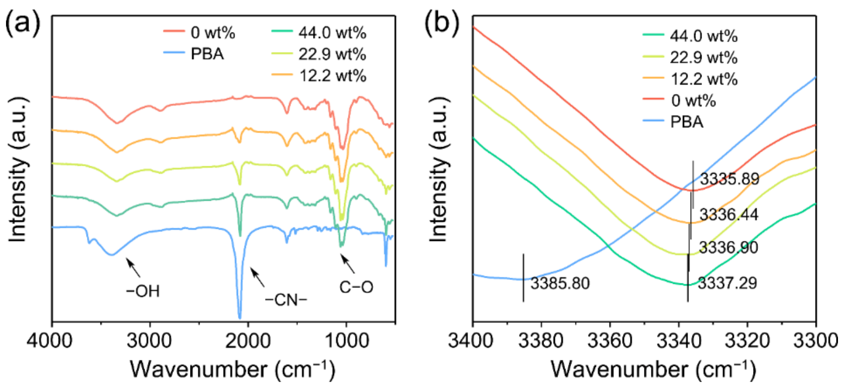

Figure S1. (a) large-scale FT-IR spectra and (b) oxhydryl functional group peaks in FT-IR spectra for PBA particles, CNF aerogel, and CNF/PBA composite aerogels through in-situ growth.

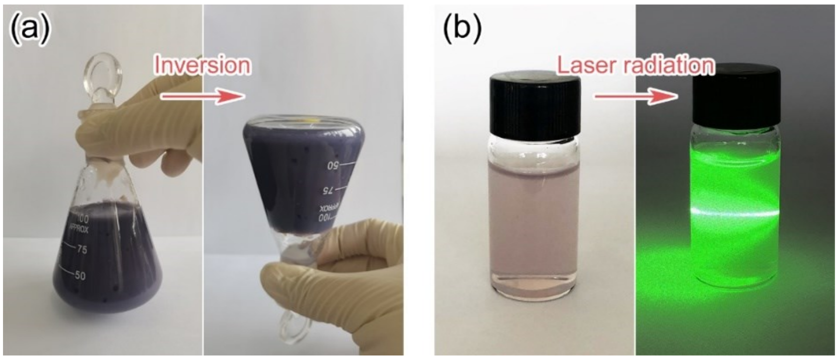

Figure S2. Photos of (a) the jelly-like PBA/CNF gels through in-situ growth; (b) the PBA/CNF dispersion at dilute conditions.

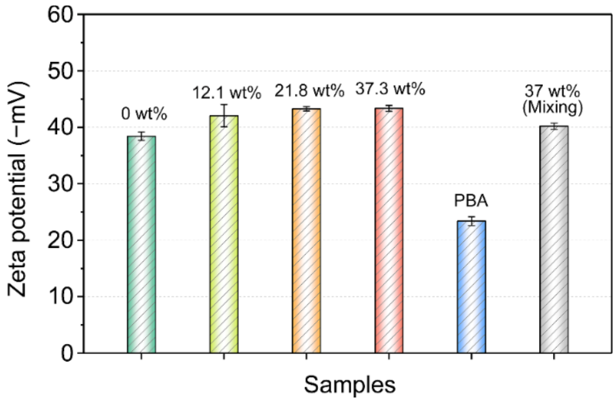

Figure S3. Zeta potentials of PBA/CNF dispersions with 0 wt%, 12.1 wt%, 21.8 wt%, and 37.3 wt% PBA growth contents. Zeta potentials of pure PBA particles, and PBA/CNF mechanical mixture with 37 wt% PBA content.

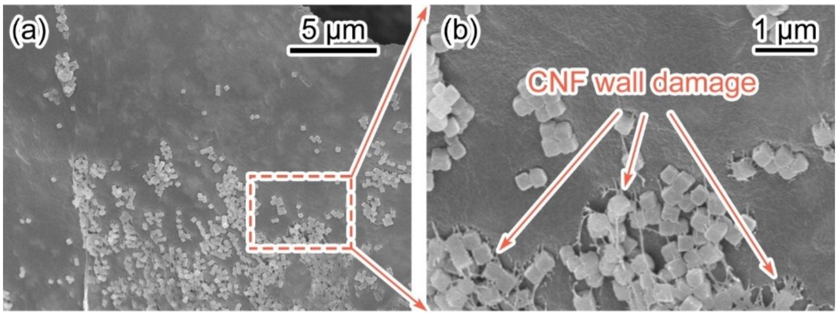

Figure S4. SEM image of PBA/CNF aerogels fabricated via a common mechanical mixing process.

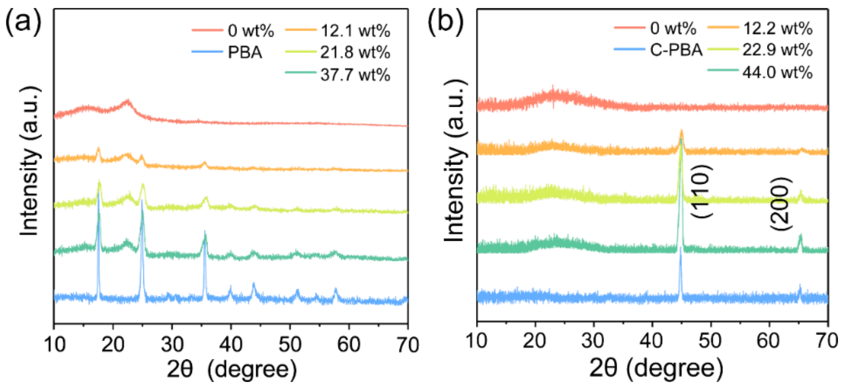

Figure S5. (a) XRD spectra of PBA particles, CNF aerogel, and CNF/PBA composite aerogels through in-situ growth. (b) XRD spectra of carbonized PBA particles, CNF-derived carbon aerogel, and CoFe/carbon aerogels.

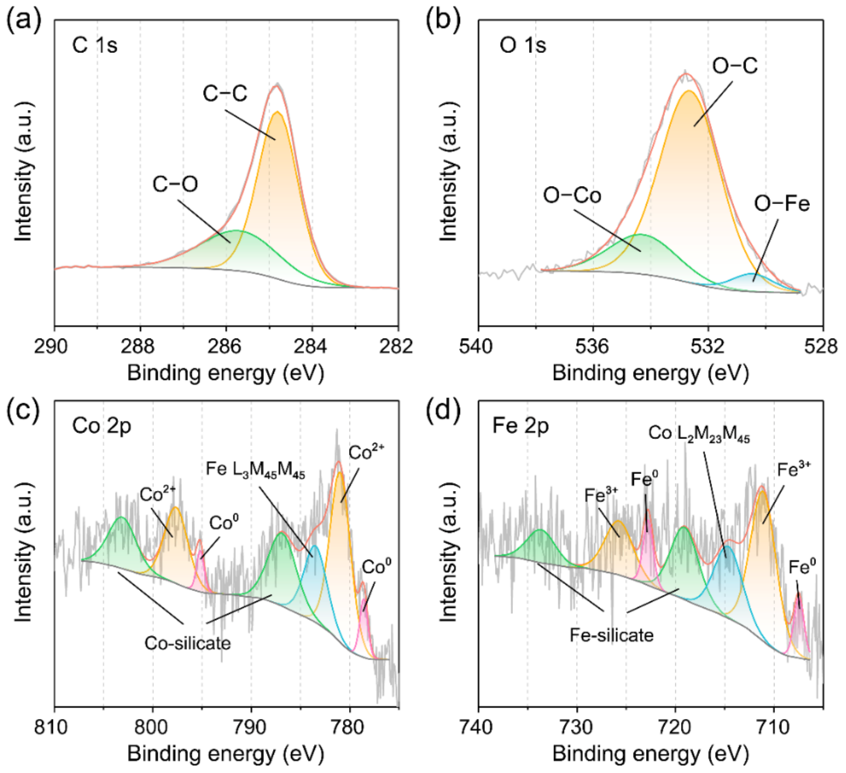

Figure S6. XPS spectra of CoFe/carbon aerogels at (a) C 1s region, (b) O 1s region, (c) Co 2p region, and (d) Fe 2p region.

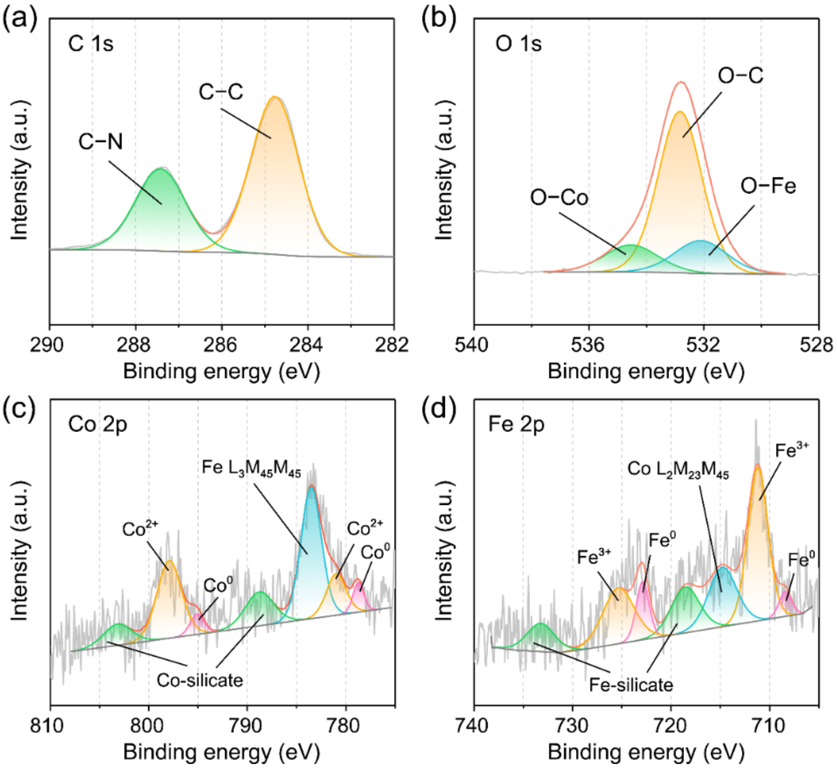

Figure S7. XPS spectra of carbonized PBA at (a) C 1s region, (b) O 1s region, (c) Co 2p region, and (d) Fe 2p region.

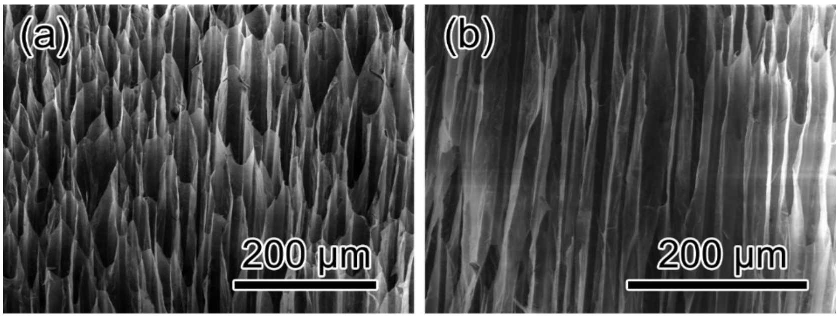

Figure S8. SEM images of CNF aerogel (a) in the top view, (b) the side view, and (c) the enlarged view.

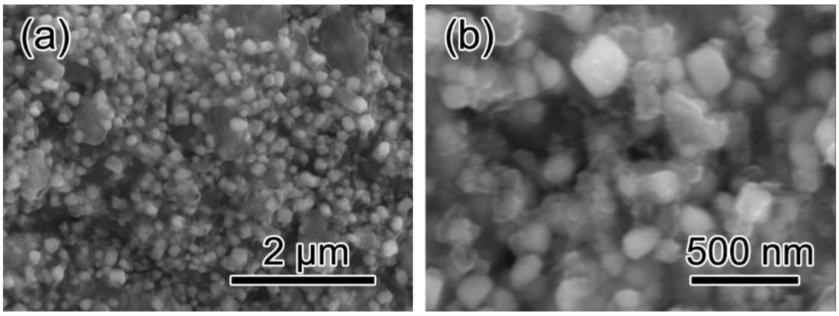

Figure S9. SEM images of CoFe/C aerogels prepared via the common mechanical mixing strategy.

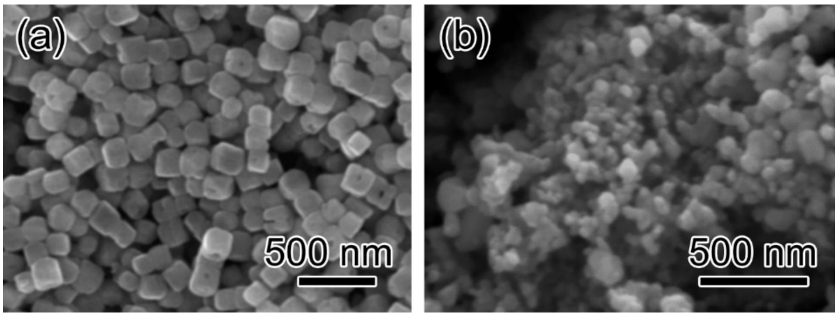

Figure S10. SEM images of (a) CoFe-PBA and (b) directly carbonized CoFe-PBA (CoFe/C).

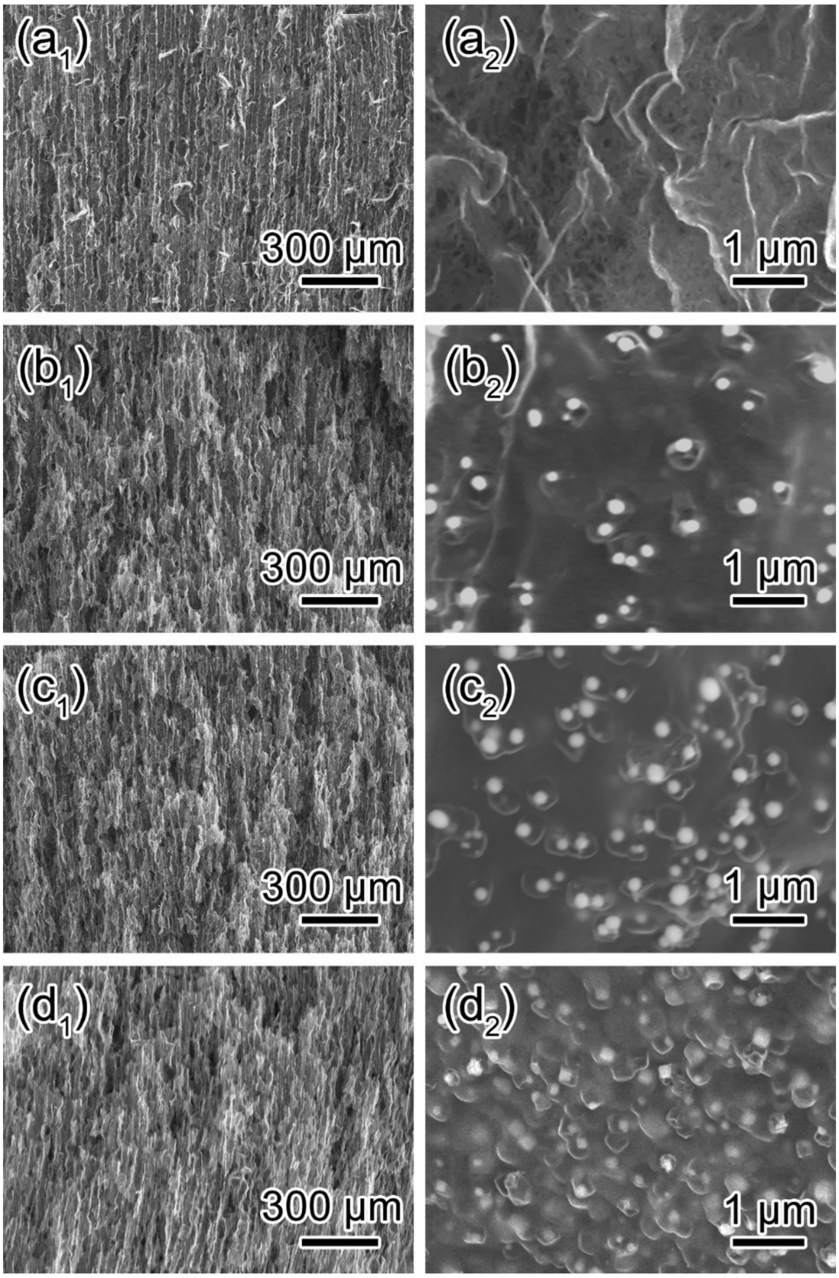

Figure S11. SEM images of (a) CNF derived carbon aerogel (A-C), and CoFe/carbon aerogels with (b) 12.2 wt%, (c) 22.9 wt%, and (d) 44.0 wt% loading contents.

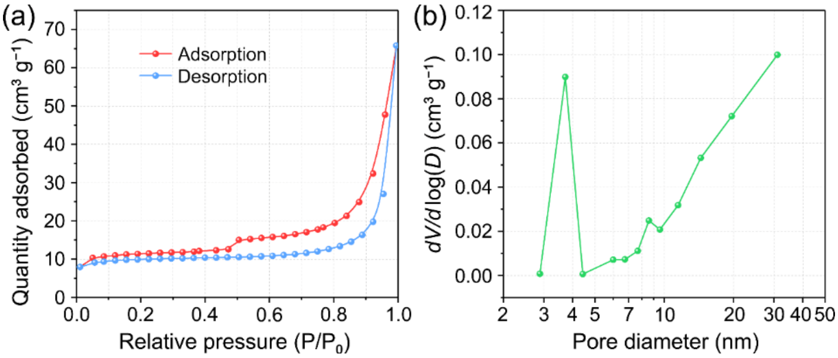

Figure S12. (a) N_2 absorption–desorption isotherm and (b) pore diameter distribution of CoFe/carbon aerogel with 22.9 wt% loading content.

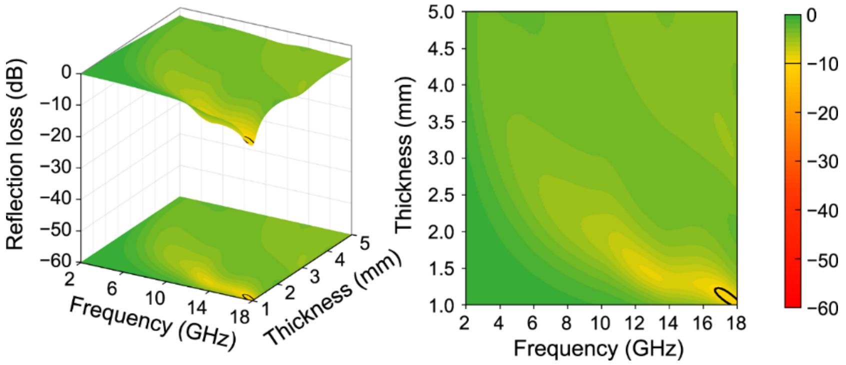

Figure S13. Three-dimensional RL representations and two-dimensional RL projection mappings of CNF-derived carbon aerogel.

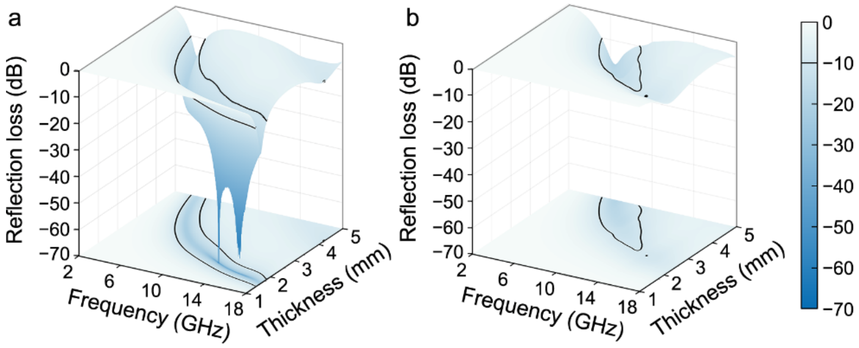

Figure S14. Three-dimensional RL representations of CoFe/carbon aerogels prepared through (a) in-situ growing PBAs on CNFs, and (b) ex-situ mechanical mixing PBA and CNFs.

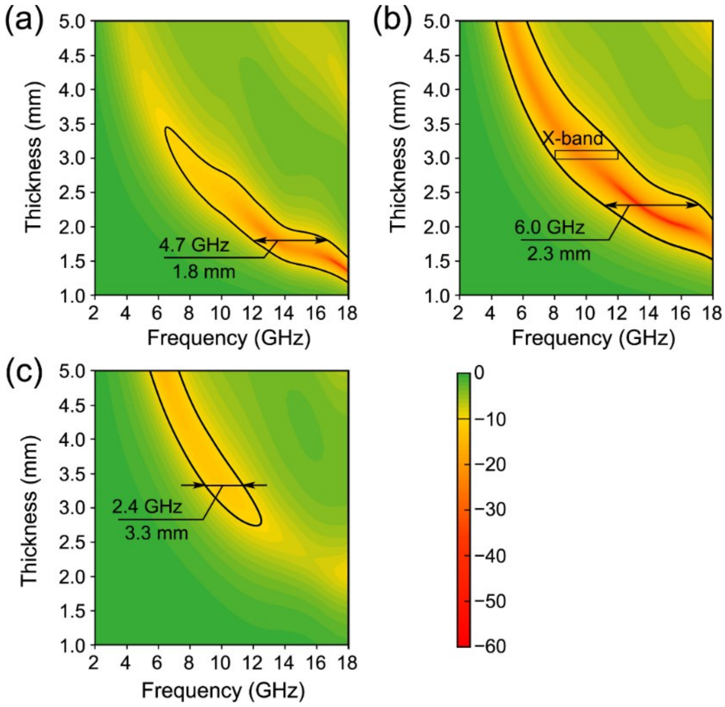

Figure S15. Two-dimensional RL projection mappings of different CoFe/carbon aerogel with (a) 12.2 wt%, (b) 22.9 wt%, and (c) 44.0 wt% loading contents.

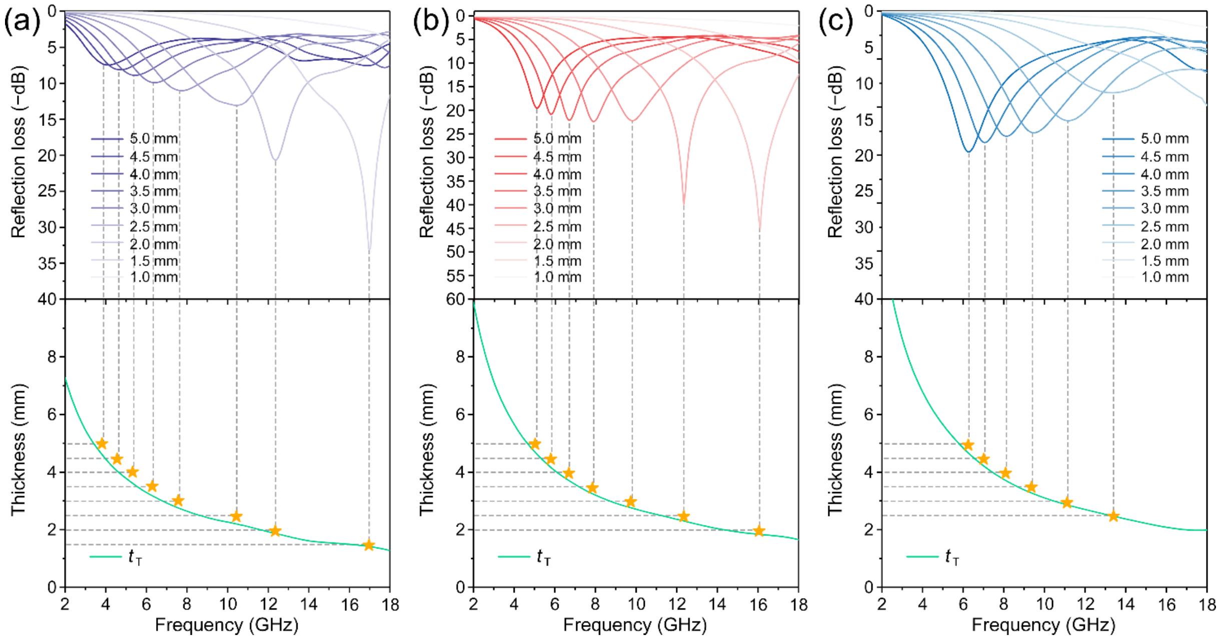

Figure S16. Reflection loss–frequency curves, relationship between matching thickness (t_M) and theoretic quarter-wavelength matching thickness (t_T) for CoFe/carbon aerogel with (a) 12.2 wt%, (a) 22.9 wt%, and (a) 44.0 wt% loading content.

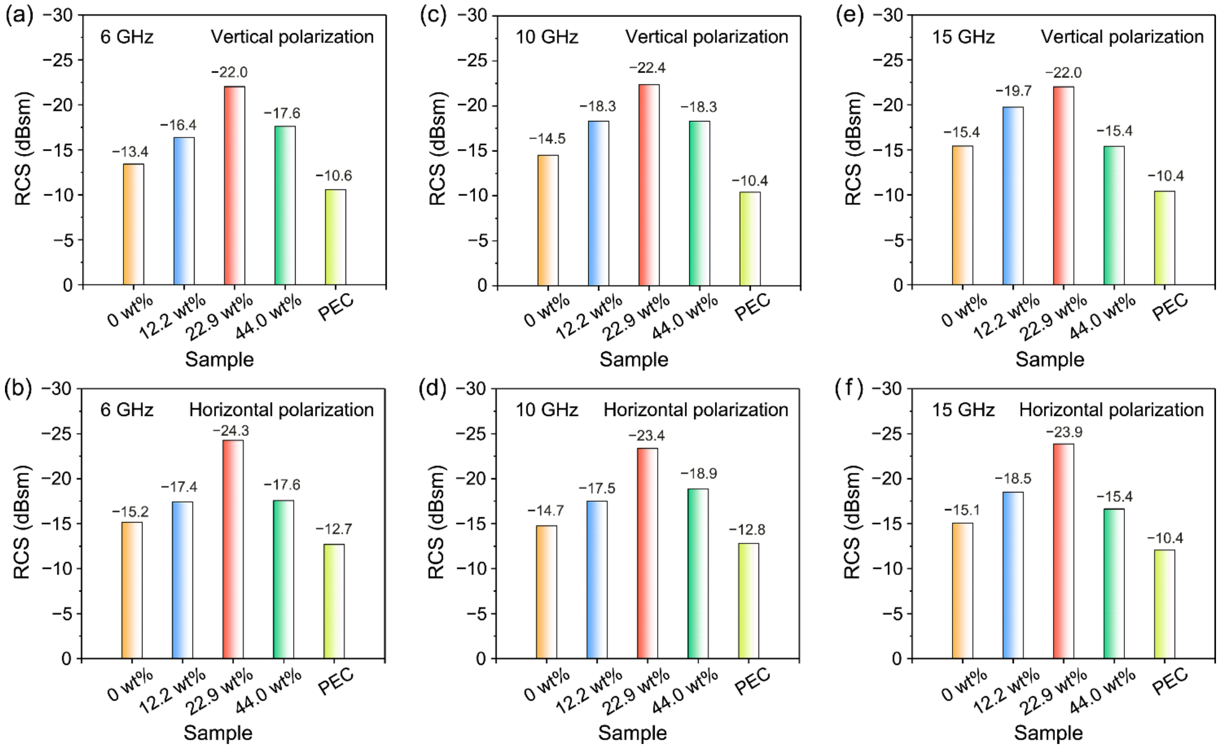

Figure S17. Average RCS values of perfect electrical conductor (PEC), CNF-derived carbon, and different CoFe/carbon aerogels (a) at 6 GHz under vertical polarization, (b) at 6 GHz under horizontal polarization, (c) at 10 GHz under vertical polarization, (d) at 10 GHz under horizontal polarization, (e) at 15 GHz under vertical polarization, and (f) at 15 GHz under horizontal polarization.

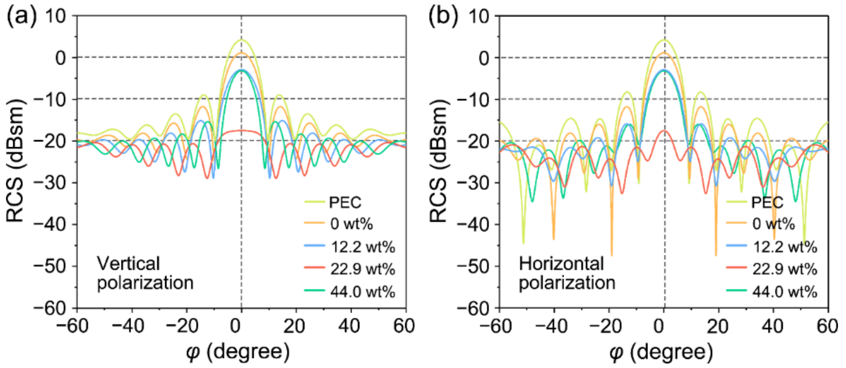

Figure S18. RCS simulation results at 6 GHz of perfect electrical conductor (PEC), CNF-derived carbon, and CoFe/carbon aerogels with 12.2 wt%, 22.9 wt%, and 44.0 wt% loading content under (e) vertical polarization and (f) horizontal polarization.

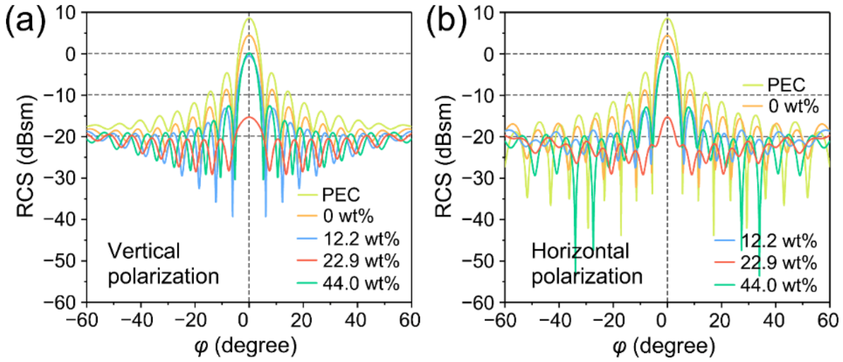

Figure S19. RCS simulation results at 10 GHz of perfect electrical conductor (PEC), CNF-derived carbon, and CoFe/carbon aerogels with 12.2 wt%, 22.9 wt%, and 44.0 wt% loading content under (e) vertical polarization and (f) horizontal polarization.

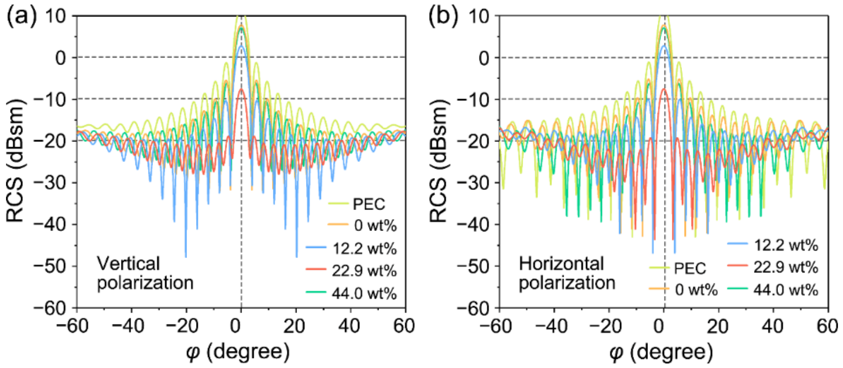

Figure S20. RCS simulation results at 15 GHz of perfect electrical conductor (PEC), CNF-derived carbon, and CoFe/carbon aerogels with 12.2 wt%, 22.9 wt%, and 44.0 wt% loading content under (e) vertical polarization and (f) horizontal polarization.

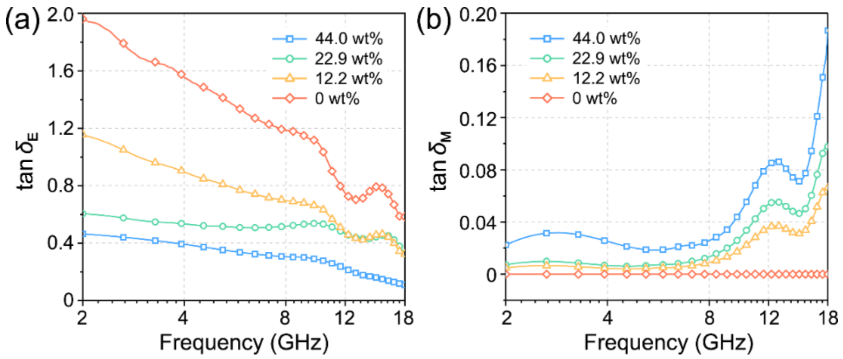

Figure S21. The (a) dielectric loss tangent and (b) magnetic loss tangent of carbon-based aerogels with 0 wt%, 12.2 wt%, 22.9 wt%, and 44.0 wt% CoFe alloy loading content.

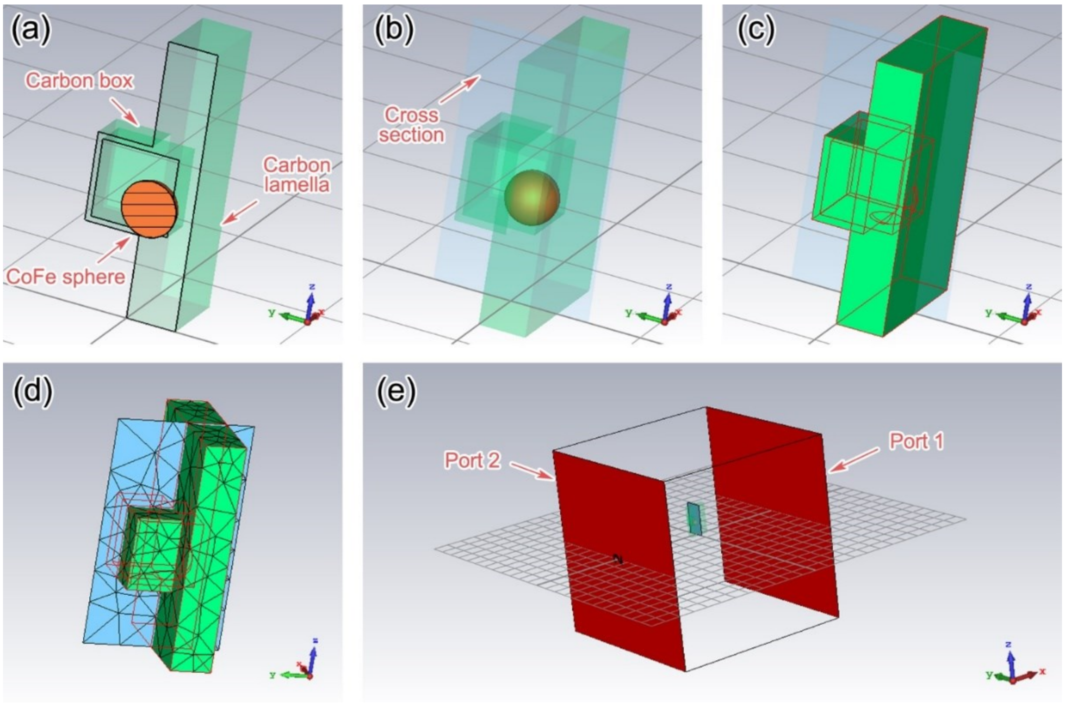

Figure S22. The constructed model of a local CoFe@C nano-capsule: (a–c) physical structure and materials, (d) mesh conditions, (e) simulated waveguide cavity.

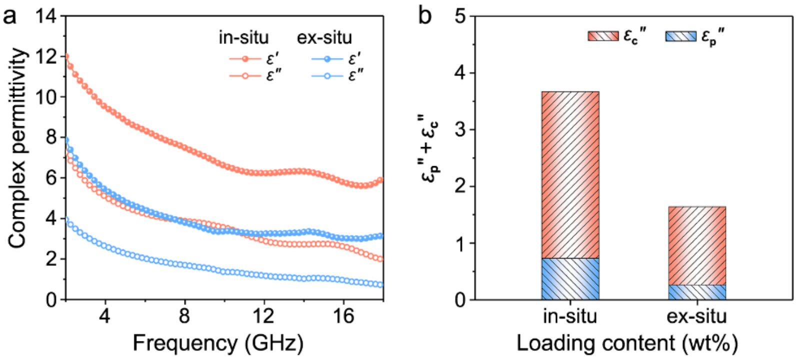

Figure S23. (a) Complex permittivity of CoFe/carbon aerogels prepared through both in-situ and ex-situ methods. (b) Fitting results about contributions of conductivity and polarization losses in CoFe/carbon aerogels prepared through both in-situ and ex-situ methods.

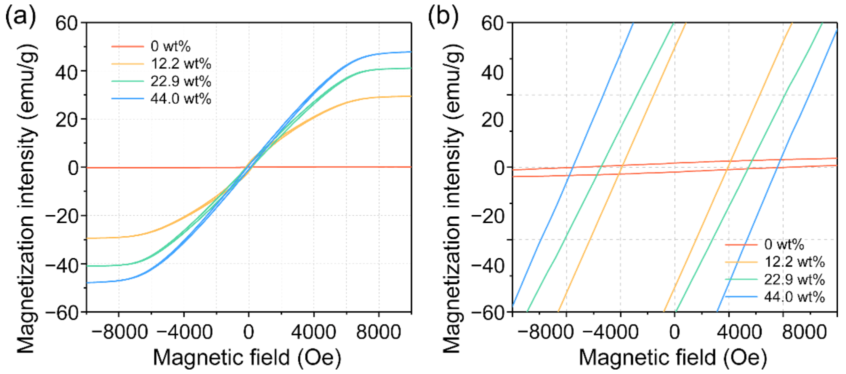

Figure S24. The electrical conductivity of CNF-derived carbon and CoFe/carbon aerogels with 12.2 wt%, 22.9 wt%, and 44.0 wt% loading content.

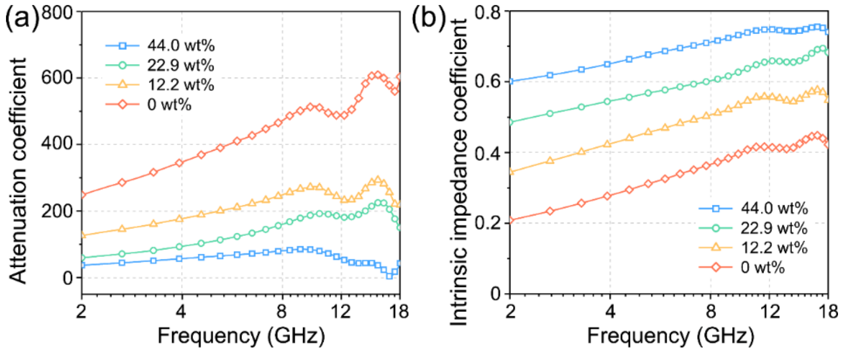

Figure S25. The (a) attenuation coefficient and (b) intrinsic impedance coefficient of carbon-based aerogels with 0 wt%, 12.2 wt%, 22.9 wt%, and 44.0 wt% CoFe alloy loading content.

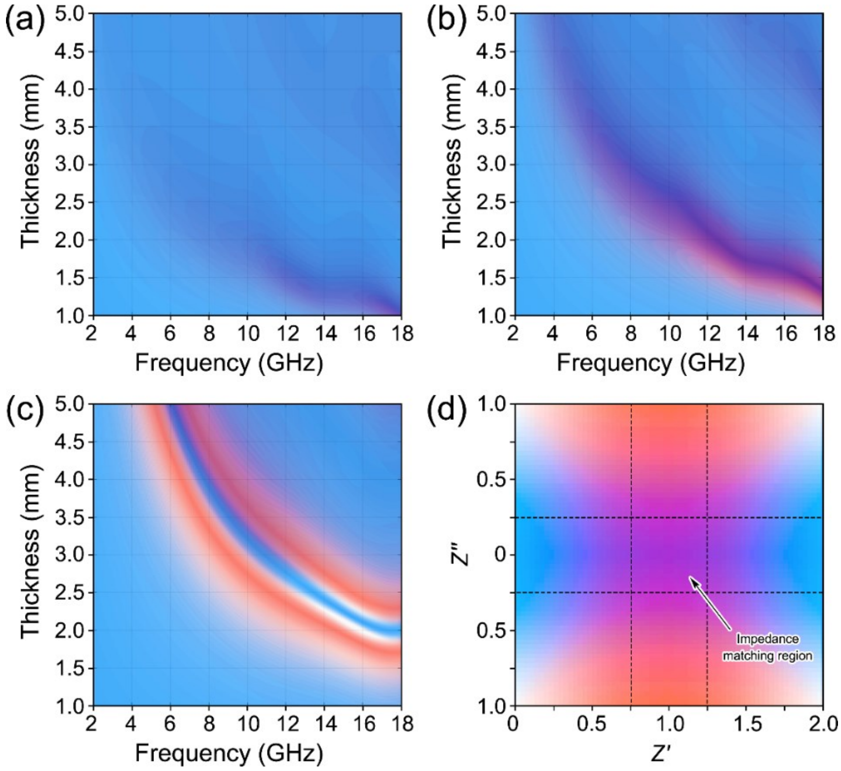

Figure S26. Two-dimensional projection drawings of *Z′* and *Z″* values *vs.* thickness and frequency for carbon-based aerogel with (a) 0 wt%, (b) 12.2 wt%, (c) 44.0 wt% CoFe alloy loading content. (d) The legend for color distribution.

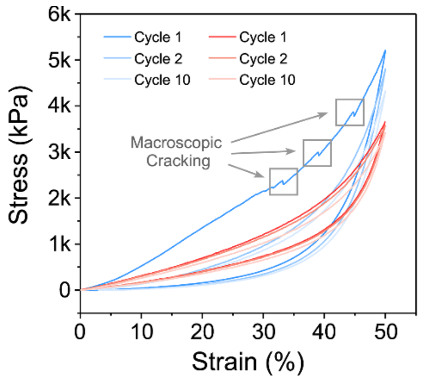

Figure S27. Compressive stress–strain curves under 50% strain for CoFe/carbon aerogels prepared through "in-situ growth" (red curves) and "ex-situ mixing" (blue curves) with the same loading content.

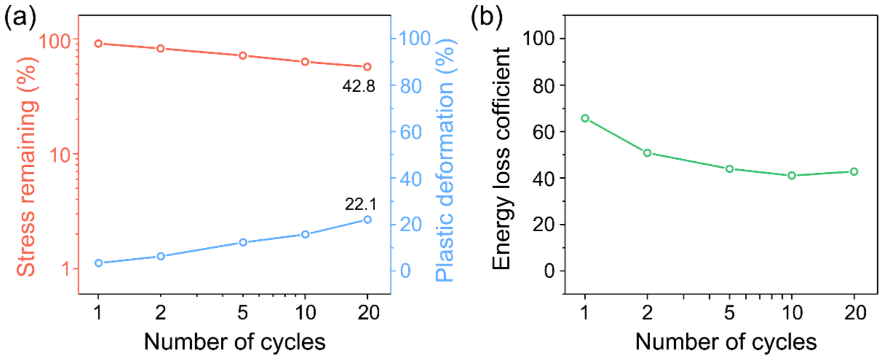

Figure S28. (a) Stress remaining, plastic deformation and (b) energy loss coefficient of CoFe/carbon aerogel during 20 cycles at 90% strain.

Section S13: Supplementary Tables (S1-S9)

Table S1. The intensity of different aerogels before/after carbonization.

Before carbonization		After carbonization	
Sample	Intensity (mg cm^{-3})	Sample	Intensity (mg cm−3)
CNF	6.4	Carbon	15.7
	8.1		18.6
PBA/CNF	10.6	CoFe/carbon	22.4
	17.7		27.3

Table S2. The volume shrinkage (($V_{initial}$–V_{final})/$V_{initial}$) of pure CNF-derived carbon and CoFe/carbon aerogels.

Sample	Volume shrinkage (%)
CNF-derived carbon	81.6
CoFe/carbon with 12.2 wt% loading	79.0
CoFe/carbon with 22.9 wt% loading	75.9
CoFe/carbon with 44.0 wt% loading	64.3

Table S3. Mass fraction of PBA in PBA/CNF aerogels, and metals in CoFe/carbon aerogels.

Sample	Mass fraction (wt.%)	Sample	Mass fraction (wt.%)		
	PBA		Co	Fe	CoFe
PBA/CNF	12.1	CoFe/carbon	7.5	4.7	12.2
	21.8		13.3	9.6	22.9
	37.3		24.9	19.1	43.9

* *The mass fraction of Co and Fe was verified by ICP.*

Table S4. The thickness of covered aerogel layer at different waveband.

Waveband	Frequency (GHz)	Thickness (mm)
C band	6	4.5
X band	10	3.0
Ku band	15	2.0

Table S5. The detail electromagnetic parameters of different aerogels.

Aerogel sample	Frequency (GHz)	ε'	tanδ_E	μ'	Tanδ_M
CNF-derived carbon	6	16.6775	1.3174	1	0
	10	12.5938	1.0929	1	0
	15	13.5097	0.7943	1	0
CoFe/carbon with 12.2 wt% loading	6	11.3015	0.7609	1.0128	0.0050
	10	8.9963	0.6549	1.0176	0.0198
	15	9.4192	0.4625	1.0014	0.0316
CoFe/carbon with 22.9 wt% loading	6	8.3379	0.5078	1.0174	0.0075
	10	6.5934	0.5374	1.0204	0.0296
	15	6.1125	0.4491	1.0034	0.0472
CoFe/carbon with 44.0 wt% loading	6	6.0426	0.3331	1.0487	0.0192
	10	5.2435	0.2846	1.0677	0.0495
	15	4.9571	0.1564	1.0067	0.0723

Table S6. Comparison of EMW absorption performances between CoFe/carbon aerogels and the reported PBA derivatives.

Materials	Minimum RL (dB)	Thickness (mm)	EAB (GHz)	Filling ratio (%)	Specific RL (dB/mm)	Ref.
NiCo@C/MnOx	−26	4	3.7	40	16.2	[1]
NiFe@C/GO	−51	2.8	3.97	30	60.7	[2]
CoFe@C	−43.5	2.5	4.3	50	34.8	[3]
La_2O_3/Co@C	−53.4	2.45	4.34	45	48.4	[4]
CoFe@C/rGO	−53	2.4	4.48	35	63.1	[5]
CoFe@C	−33	3.3	4.6	40	25.0	[6]
NiCo@void@C	−50.97	2.05	5.2	40	62.1	[7]
CoFe@C/C	−67.8	2.0	5.3	50	67.8	[8]
NiFe@C	−41	1.65	6.0	40	22.0	[9]
Co@C/CNF	−29.05	4.2	6.35	20	34.5	[10]
FeMnOx@C	−25	2.5	6.7	33.3	30.0	[11]
CoFe@C	−50.77	2.4	7.2	40	52.8	[12]
NiCo@C	−68.4	2.14	5.8	40	79.9	[13]
CoNi@C/C	−56.78	2.0	5.51	30	94.6	[14]
NiCoFe@C	−57.5	2.0	5.44	16.7	172.1	[15]
CoFe/C aerogel	−64.0	2.0	6.0	2.2	1454.5	This work

Table S7. Comparison of EMW absorption performances between CoFe/carbon aerogels and other reported carbon-based aerogels.

Materials	Minimum RL (dB)	Thickness (mm)	EAB (GHz)	Filling ratio (%)	Specific RL (dB/mm)	Ref.
SiC/MDCF	−18.41	3.65	1.64	40	12.6	[16]
Fe_3O_4/C	−50.96	5.0	3.82	40	25.4	[17]
Ni-SA/rGO	−49.46	2	3.12	25	98.9	[18]
CNT/PA66	−44.3	3	3.5	10.5	140.6	[19]
Co@C/CG-rGO	−45.02	1.5	4.02	30	100.0	[20]
Ni/NiO-NCA	−41.9	1.5	4.24	50	55.8	[21]
Fe_3O_4/ZIF-67/WA	−23.4	1.5	4.9	14.7	106.1	[22]
$MAPbI_3$/CNTs	−57.71	1.96	6.32	40	73.6	[23]
CNT/PPy	−28.34	2.5	6	10	113.3	[24]
Cellulose-C	−51.24	4.45	7.68	20	57.5	[25]
TiO_2/$Ti_3C_2T_x$/rGO	−65.3	3.5	4.3	10	186.5	[26]
CeO_2/C	−56.04	1.9	5.28	20	147.4	[27]
N-rGO/CNT	−46.3	1.4	4.2	15	220.4	[28]
Ni/C	−57.0	2.0	4.0	10	285.0	[29]
rGO/CNT/$ZnFe_2O_4$	−52.6	1.7	5.1	10	309.4	[30]
Co/C/PPy	−44.76	2.0	6.56	10	223.8	[31]
CNS/Co@CNT	−27.5	2.5	7.4	5	220.0	[32]
NiAl-LDH/G	−41.5	1.4	4.4	7	423.5	[33]
Ni/rGO	−51.19	1.9	6.32	2	1347.1	[34]
CoFe/C aerogel	−64.0	2.0	6.0	2.2	1454.5	This work

Table S8. The simulation parameters of electric-field distribution by Ansys HFSS

Model Parameters (nm)				
**Shape**	**Materials**	**Height**	**Width**	**Thickness**
cuboid	carbon	1000	600	180
**Shape**	**Materials**	**Inner edge length**	**Outer edge length**	**Thickness**
cube box	carbon	320	280	20
**Shape**	**Materials**	**radius**		
sphere	CoFe metal	100		

Material Parameters					
Materials	Permittivity	Bulk Conductivity (S m^{-1})	Dielectric Loss Tangent	Measured Frequency (GHz)	Mass Density (kg m^{-3})
CoFe metal	30.0	106	/	/	8400
Carbon	12.59	/	1.126	10.0	2,250

Table S9. The stress remaining and plastic deformation of CoFe/carbon aerogel compared with other polymer-derived carbon aerogels.

Materials	Strain (%)	Cycle numbers	Stress remaining (%)	plastic deformation (%)	Ref.
Cellulose-derived carbon	50	10^3	85	6.7	[35]
Cellulose-derived carbon	50	5×10^2	81	4	[36]
rGO/cellulose-derived carbon	50	10^2	80	2.9	[37]
Cellulose-derived carbon	50	5×10^2	76.9	1.27	[38]
konjac glucomannan-derived carbon	50	10^3	75	4.3	[39]
rGO/cellulose-derived carbon	50	5×10^2	75.3	5	[40]
rGO/oil-derived carbon	50	10^1	69	5	[41]
Cellulose-derived carbon	60	10^3	72	15.3	[42]

Section S14: Supplementary References

[1] J. Gao, H. Wang, Y. Zhou, Z. Liu, Y. He, *J. Alloys Compd.* **2022**, 892, 162151.

[2] Z. Yang, H. Lv, R. Wu, *Nano Res.* **2016**, 9, 3671–3682.

[3] X. Zeng, B. Yang, L. Zhu, H. Yang, R. Yu, *RSC Adv.* **2016**, 6, 105644.

[4] Z. Yao, F. Liu, S. Xu, X. Zhang, C. Rong, Z. Xiong, J. Yuan, Y. Yu, X. Zhu, H. Yu, *Carbon* **2022**, 196, 763–773.

[5] S. Wei, T. Chen, Z. Shi, S. Chen, *J Colloid Interface Sci* **2022**, 610, 395.

[6] D. Liu, R. Qiang, Y. Du, Y. Wang, C. Tian, X. Han, *J. Colloid Interface Sci.* **2018**, 514, 10–20.

[7] C. Li, X. Qi, X. Gong, Q. Peng, Y. Chen, R. Xie, W. Zhong, *Nano Res.* **2022**, 15, 6761–6771.

[8] F. Wang, N. Wang, X. Han, D. Liu, Y. Wang, L. Cui, P. Xu, Y. Du, *Carbon* **2019**, 145, 701–711.

[9] X. Liang, G. Wang, W. Gu, G. Ji, *Carbon* **2021**, 177, 97–106.

[10] P. Yi, X. Zhang, L. Jin, P. Chen, J. Tao, J. Zhou, Z. Yao, *Chem. Eng. J.* **2022**, 430, 132879.

[11] P. Miao, J. Chen, Y. Tang, K.-J. Chen, J. Kong, *Sci. China Mater.* **2020**, 63, 2050–2061.

[12] S. Wei, Z. Shi, X. Li, T. Chen, H. Gao, S. Chen, *J. Alloys Compd.* **2022**, 910, 164861.

[13] L. Cui, Y. Wang, X. Han, P. Xu, F. Wang, D. Liu, H. Zhao, Y. Du, *Carbon* **2021**, 174, 673–682.

[14] X. Wu, W. Ma, J. Xu, P. He, Y. Du, Y. Zhang, P. Zuo, H. Qi, Q. Zhuang, *ACS Appl. Nano Mater.* **2022**, 5, 7300–7311.

[15] Y. Wang, Z. Pang, H. Xu, C. Li, W. Zhou, X. Jiang, L. Yu, *J. Colloid Interface Sci.* **2022**, 620, 107–118.

[16] X. Ye, Z. Chen, S. Ai, B. Hou, J. Zhang, X. Liang, Q. Zhou, H. Liu, S. Cui, *ACS Sustainable Chem. Eng.* **2019**, 7, 2774–2783.

[17] Q. Zhu, X. Zhang, X. Wang, X. Wu, Z. Zhang, J. Shen, *J. Magn. Mag. Mater.* **2022**, 541, 168511.

[18] B. Suo, X. Zhang, X. Jiang, F. Yan, Z. Luo, Y. Chen, *Chinese Phys. Lett.* **2022**, 39, 045201.

[19] X. Fu, Y. Guo, L. Guan, J. Liu, *Mater. Lett.* **2019**, 247, 147–150.

[20] J. Xu, X. Zhang, Z. Zhao, H. Hu, B. Li, C. Zhu, X. Zhang, Y. Chen, *Small* **2021**, 17, 2102032.

[21] L. Wang, M. Liu, G. Wang, B. Dai, F. Yu, J. Zhang, *J. Alloys Compd.* **2019**, 776, 43–51.
[22] L. Xu, Y. Xiong, B. Dang, Z. Ye, C. Jin, Q. Sun, X. Yu, *Mater. Design* **2019**, 182, 108006.
[23] Z. Zhang, Z. Xiong, Y. Yao, D. Wang, Z. Yang, P. Zhang, Q. Zhao, W. Zhou, *Adv. Funct. Mater.* **2022**, 32, 220653.
[24] K. Zhang, A. Xie, F. Wu, W. Jiang, M. Wang, W. Dong, *Mater. Res. Express* **2016**, 3, 055008.
[25] T. Bai, Y. Guo, H. Liu, G. Song, D. Zhang, Y. Wang, L. Mi, Z. Guo, C. Liu, C. Shen, *J. Mater. Chem. C* **2020**, 8, 5191–5201.
[26] Y. Tong, M. He, Y. Zhou, S. Nie, X. Zhong, L. Fan, T. Huang, Q. Liao, Y. Wang, *ACS Sustainable Chem. Eng.* **2018**, 6, 8212–8222.
[27] X. Huang, X. Liu, Z. Jia, B. Wang, X. Wu, G. Wu, *Adv. Compos. Hybrid Mater.* **2021**, 4, 1398–1412.
[28] Z. Wan, R. Shu, J. Zhang, Y. Wu, *Diam. Relat. Mater.* **2021**, 112, 108245.
[29] H.-B. Zhao, Z.-B. Fu, X.-Y. Liu, X.-C. Zhou, H.-B. Chen, M.-L. Zhong, C.-Y. Wang, *Ind. Eng. Chem. Res.* **2017**, 57, 202–211.
[30] N. Li, R. Shu, J. Zhang, Y. Wu, *J. Colloid Interface Sci.* **2021**, 596, 364–375.
[31] X. Sun, X. Lv, M. Sui, X. Weng, X. Li, J. Wang, *Materials* **2018**, 11, 781.
[32] Y. Fei, X. Wang, M. Yuan, M. Liang, Y. Chen, H. Zou, *Ind. Eng. Chem. Res.* **2022**, 61, 1684–1693.
[33] X. Xu, S. Shi, Y. Tang, G. Wang, M. Zhou, G. Zhao, X. Zhou, S. Lin, F. Meng, *Adv. Sci.* **2021**, 8, 2002658.
[34] K. Cao, X. Yang, R. Zhao, W. Xue, *ACS Appl. Mater. Interfaces* **2023**, 15, 9685–9696.
[35] H. Lai, H. Zhou, Y. Hu, G. Shi, Z. Chen, L. Zhong, M. Zhang, *ACS Sustainable Chem. Eng.* **2021**, 9, 9761–9769.
[36] L. Li, T. Hu, H. Sun, J. Zhang, A. Wang, *ACS Appl. Mater. Interfaces* **2017**, 9, 18001–18007.
[37] X. Zhang, J. Zhao, X. He, Q. Li, C. Ao, T. Xia, W. Zhang, C. Lu, Y. Deng, *Carbon* **2018**, 127, 236–244.
[38] Q. Wang, T. Xia, X. Jia, J. Zhao, Q. Li, C. Ao, X. Deng, X. Zhang, C. Lu, *Carbohydr. Polym.* **2020**, 245, 116554.
[39] Y. Si, X. Wang, C. Yan, L. Yang, J. Yu, B. Ding, *Adv. Mater.* **2016**, 28, 9512–9518.
[40] D. Liao, Y. Wang, P. Xie, C. Zhang, M. Li, H. Liu, L. Zhou, C. Wei, C. Yu, Y. Chen, *J. Colloid Interface Sci.* **2022**, 628, 574–587.

[41] S. Barg, F. M. Perez, N. Ni, P. V. Pereira, R. C. Maher, E. G. Tunon, S. Eslava, S. Agnoli, C. Mattevi, E. Saiz, *Nat. Commun.* **2014**, 5, 4328.

[42] J. Zhang, B. Li, L. Li, A. Wang, *J. Mater. Chem. A* **2016**, 4, 2069–2074.
